# Photopharmacologic Vision Restoration Reduces Pathological Rhythmic Field Potentials in Blind Mouse Retina

**DOI:** 10.1038/s41598-019-49999-w

**Published:** 2019-09-19

**Authors:** Katharina Hüll, Tyler Benster, Michael B. Manookin, Dirk Trauner, Russell N. Van Gelder, Laura Laprell

**Affiliations:** 1 Center for Integrated Protein Science Munich and Department of Chemistry, Ludwig-Maximilians-Universität München, Munich, Germany; 2 Department of Chemistry, New York University, 100 Washington Square East, New York, New York, 10003-6699 United States; 3 Department of Ophthalmology, University of Washington School of Medicine, Seattle, Washington USA; 4 Present Address: Neurosciences Graduate Program, Stanford University, Stanford, CA 94305 USA; 5 Departments of Biological Structure and Pathology, University of Washington School of Medicine, Seattle, Washington USA; 6 Department of Synaptic Physiology, Center for Molecular Neurobiology, ZMNH, University Medical Center Hamburg-Eppendorf, Hamburg, Germany

**Keywords:** Neurophysiology, Translational research

## Abstract

Photopharmacology has yielded compounds that have potential to restore impaired visual responses resulting from outer retinal degeneration diseases such as retinitis pigmentosa. Here we evaluate two photoswitchable azobenzene ion channel blockers, DAQ and DAA for vision restoration. DAQ exerts its effect primarily on RGCs, whereas DAA induces light-dependent spiking primarily through amacrine cell activation. Degeneration-induced local field potentials remain a major challenge common to all vision restoration approaches. These 5–10 Hz rhythmic potentials increase the background firing rate of retinal ganglion cells (RGCs) and overlay the stimulated response, thereby reducing signal-to-noise ratio. Along with the bipolar cell-selective photoswitch DAD and second-generation RGC-targeting photoswitch PhENAQ, we investigated the effects of DAA and DAQ on rhythmic local field potentials (LFPs) occurring in the degenerating retina. We found that photoswitches targeting neurons upstream of RGCs, DAA (amacrine cells) and DAD (bipolar cells) suppress the frequency of LFPs, while DAQ and PhENAQ (RGCs) had negligible effects on frequency or spectral power of LFPs. Taken together, these results demonstrate remarkable diversity of cell-type specificity of photoswitchable channel blockers in the retina and suggest that specific compounds may counter rhythmic LFPs to produce superior signal-to-noise characteristics in vision restoration.

## Introduction

Outer retinal degenerative diseases, such as retinitis pigmentosa and age-related macular degeneration, affect millions of people worldwide. While in these diseases the photoreceptor cell layer degenerates, the rest of the retinal circuitry remains largely intact, undergoing slow remodeling. Restoration of impaired vision is a long-sought goal^1^.

**Figure 1:**
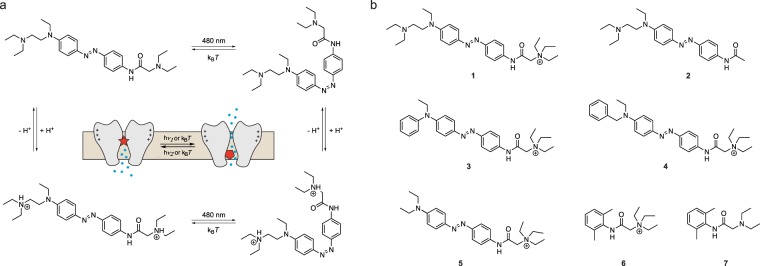
Structures of photochromic ion channel blockers. (**a**) DAD is a non-permanently charged third generation photoswitch applied for vision restoration. DAD can exist in a deprotonated or protonated form. The uncharged state should be plasma-membrane permeable, enabling efficient loading into retinal neurons. Irradiation with blue or white light converts DAD to its less stable *cis*-isomer, which quickly relaxes back to *trans* in darkness. Inset: Schematic view of the photoswitch blocking mechanism (Adapted from^6^). (**b**) Molecular structures of DAQ (**1**), DAA (**2**), PhENAQ (**3**), BENAQ (**4**), DENAQ (**5**), QX-314 (**6**) and lidocaine (**7**).

Recently, we and others have pursued photopharmacological approaches for vision restoration, utilizing small freely-diffusible azobenzene photoswitches that target intrinsic neuronal receptors in different retinal cell types^2,3^. Photoswitches can be reversibly switched between two configurations, a thermodynamically stable *trans*- and a less stable *cis*-configuration, using light of visible wavelengths (Fig. 1). Whereas in one configuration the photoswitch blocks its target channel, the other configuration leads to release of the photoswitch from the pore (Fig. 1 inset). By switching between the two configurations using visible (blue/white) light and darkness, light-dependent ganglion cell firing can be restored in the remaining circuitry of the blind retina^2,4–7^.

Retinal reanimation using photopharmacology is a promising approach for the restoration of vision. However, being a pharmacological approach, the optimization of cell specificity, membrane permeability, and kinetics becomes a major challenge in further development. Cell specificity of photoswitches becomes especially important when considering the progression of retinal degeneration. While in early stages of disease the intermediate layers of the retina (i.e. bipolar and amacrine cells) are still in place and can be pharmacologically targeted; in late stage retinas, death of bipolar and amacrine cells leads to remodeling of the retina. Therefore, at late stage retinal degeneration targeting RGCs might be the only option^8–10^. Having a toolbox at hand that targets different stages of disease becomes therefore desirable.

The onset of photoreceptor cell loss is accompanied by strong spontaneous oscillatory activity in the retina arising in the bipolar-amacrine cell network^11,12^. This oscillatory activity represents a major challenge to all vision restoration approaches as it increases background firing rate and may overlay the stimulated response, thereby reducing the signal-to-noise ratio. In mouse models for retinal degeneration, e.g. *rd1* and *rd10*, spontaneous oscillations become evident about the age of first eye opening^13^. In *rd10* mice, a mouse model for slow retinal degeneration, rhythmic local field potentials (LFPs) occur first at frequencies around ~5 Hz and increase in frequency with disease progression. In *rd1* mice, disease onset occurs earlier, and strong 5 and 10 Hz LFPs are observed a few weeks after birth^14^. LFPs coincide with rhythmic bursting activity in RGCs in both *rd1* and *rd10* mouse models for retinal degeneration^14,15^.

One approach that has been investigated for reduction of LFPs is the application of gap junction blockers, such as meclofenamic acid (MFA)^16^. Blocking gap junctions between bipolar and AII amacrine cells lead to the reduction of this pathological hyperactivity and underlying LFPs. When applied in *rd1* retinas rescued with ganglion cell expression of ChR2 this in turn lead to a significant increase in signal-to-noise of RGC output. However, this strategy has its limitations: MFA is a non-specific agent (it was originally approved as a COX inhibitor) and influences the entire retinal circuitry, thus limiting its use to approaches that target RGCs^17^.

To date, apart from the third-generation photoswitch DAD, which targets bipolar cells, most studied fast-relaxing photochromic open-channel blockers primarily target retinal ganglion cells (RGCs)^3–5^. Here we extend the photopharmacological toolbox for vision restoration approaches with two novel compounds and show that targeting cells upstream from RGCs leads to a reduction in LFPs. Furthermore, we demonstrate that photoswitches that target bipolar and amacrine cells suppress oscillatory activity when activated by light, even well after onset of strong ~5 Hz oscillations.

Both photoswitches are derivatives of the sodium channel blockers lidocaine or QX-314 (Fig. 1B). It has been previously shown that azobenzene-derivatives of QX-314 do not primarily target sodium channels, but exert their effects mainly through voltage-gated potassium channels^18^. Since potassium channels are widely expressed in a variety of cell types, and particularly in neurons, these photoswitches should in principle target all cells in the remaining retinal circuitry of a blind mouse. However, we recently discovered that the non-permanently charged lidocaine derivative DAD primarily targets bipolar cells in the degenerating retina, but not in the wild-type or in the morphologically intact but blind retina and has only negligible effects on amacrine or retinal ganglion cells^6^. The permanently charged QX-314 derivatives DENAQ and BENAQ, in contrast, primarily target RGCs, relying on an uptake mechanism through dilated P2X channels^3,5^. In the current study we developed intermediate compounds between DAD and BENAQ to study their functionality in the degenerating retina and to address the optimization process for photoswitch pharmacology in respect to target cell type and effect on LFPs.

## Results

### Open-channel blocker design, synthesis and characterization

To better understand the pharmacology and mechanism-of-action of open-channel blockers, we synthesized derivatives of the soluble azobenzene DAD, named DAQ and DAA (Scheme S1 and 2)^6^. In an attempt to change characteristics of the molecules such as membrane permeability, we modified both sides of the azobenzene in DAD. In comparison to previously published photoswitches applied in blind retina, DAD has two possible, but structurally different pharmacophores (Fig. 1). Under physiological conditions both amines in DAD are protonated^19^. Although not initially considered as a pharmacophore, we wanted to investigate if the common motif in DAD and DAQ, the aminoethylaniline functionality alone (truncated blocker: DAA, Fig. 1B) can induce light-dependent responses in blind retina and how it influences LFPs. We also sought to determine if the presence of a permanent charge (DAQ) is sufficient to influence cell subtype selectivity. This effect is suggested by the previously studied charged open-channel blockers DENAQ and BENAQ, which are RGC-specific (3, 5), but otherwise share similarities with the non- permanently charged DAD.

DAQ and DAA were synthesized in 6 steps using a divergent approach, following the synthetic route of DAD. UV-Vis characterization showed similar properties for all three compounds (Fig. S1): DAD, DAQ and DAA could be isomerized by blue light and are fast-relaxing in the dark. All compounds were prepared as HCl salts to improve solubility. PhENAQ, a ‘second generation’ charged photoswitch, was synthesized for comparison as previously described^18^.

DAD, DAQ and DAA were initially characterized in layer 2/3 cortical neurons of acute mouse brain slice preparations. Like DAD, DAQ blocks and unblocks potassium channels in cortical neurons^6^. The optimal switching wavelengths were determined to be 460 nm and 540 nm for unblock and block, respectively, and the kinetics of block and unblock are comparable to those of DAD (τ_on_ = 16.0 ± 0.49 ms, τ_off_ = 93.5 ± 2.12 ms^6^) (Fig. S2). In addition, sodium channel currents in layer 2/3 neurons of acute brain slice preparations were not affected by DAQ application, similar to what has been shown for DAD (Fig. S2G,H). Taken together, DAQ’s photoswitching properties closely resemble those of DAD in acute brain slice preparation.

Characterization of DAA in murine brain slice also showed light-induced K_V_-channel currents in depolarized cortical cells upon blue light illumination (Fig. S3). In addition, the wavelength sensitivity was comparable to DAD and DAQ with a maximum near 460 nm (consistent with the respective UV-Vis spectra S1). The major difference between DAD, DAQ and DAA in layer 2/3 neurons was detected in the photoswitch off-kinetics. Whereas DAQ has a relatively fast τ_off_ similar to DAD (τ_off(DAD_) = 72.1 ± 8.7 ms^6^), DAA was significantly slower when illuminated with 540 nm light (τ_off_ = 204 ± 20.3 ms) (Fig. S3F).

In summary, all three molecules, DAD, DAQ and DAA act as open-channel blocker in their *trans* state and unblock the pore upon isomerization with blue light to *cis*, making all three compounds suitable candidates for application to vision restoration of degenerated retina.

### DAD targets bipolar cells and reduces local field potentials in the degenerating retina

**Figure 2:**
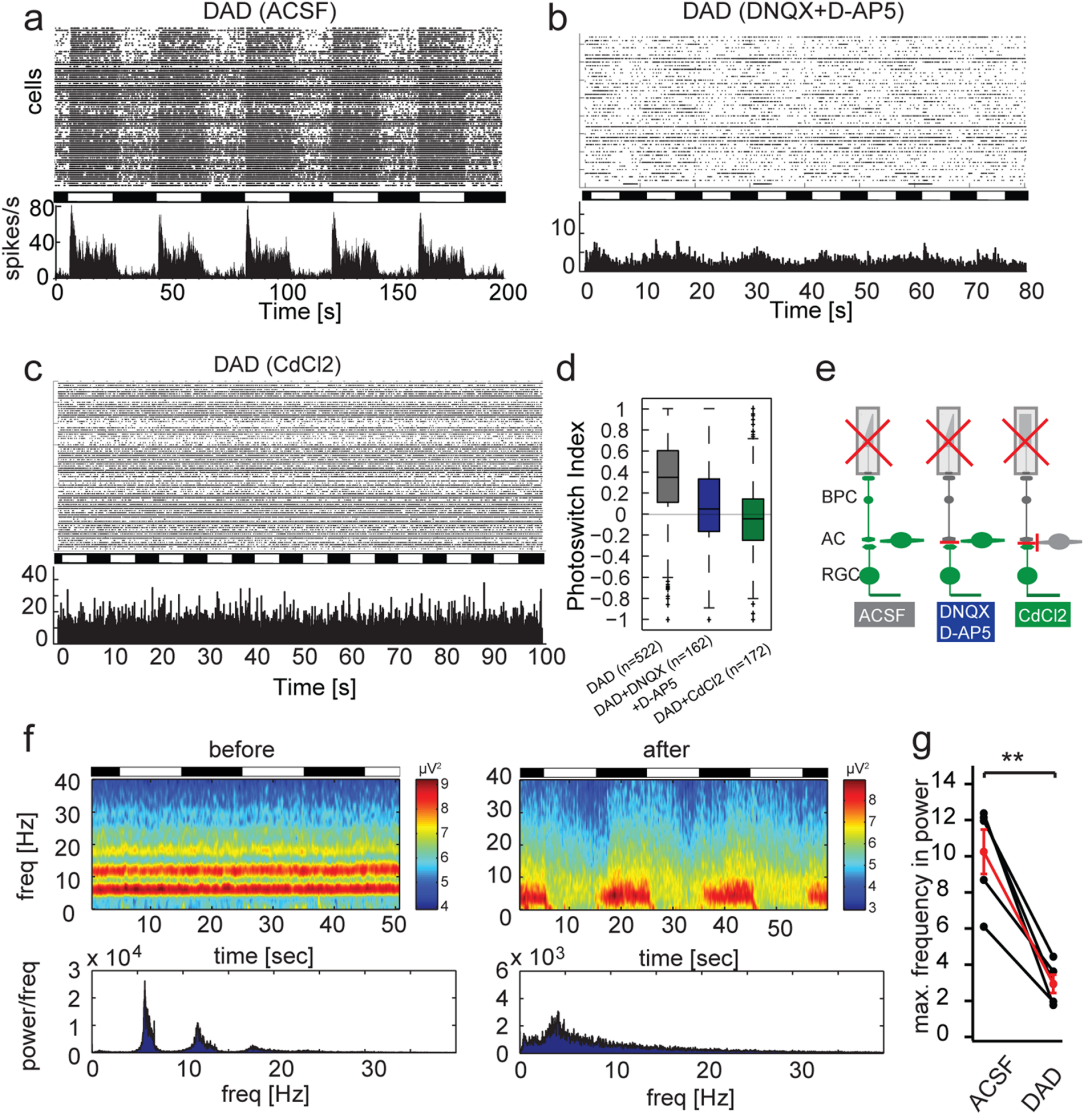
DAD suppresses LFPs in degenerating retina. (**a**) Raster plot and histogram of representative MEA recording in an *rd1/rd1*,*Opn4*^−/−^ retina after treatment with 200 µM DAD. (**b**) Same as in A, with additional application of DNQX and D-AP5. (**c**) Same as in A, with additional application of CdCl_2_. (**d**) Quantification of light responses using the photoswitch index. (**e**) Graphical overview of pharmacological experiments. (**f**) Power spectrum of rhythmic firing due to oscillatory local field potentials before and after application of DAD. Heat plot represents spectral power at noted frequency in Hz. (**g**) Quantification of maximal frequency response before and after application of DAD (average intensities of complete time range). Maximum frequency with significant power is defined by the peak of the highest frequency reaching a threshold of 4 SD. Each data point refers to the power average of local field potentials in one retina. (n = 4 retinas). The average change in power is depicted in red with error bars (mean ± SEM).

DAD (Fig. 1A) is a non-permanently charged, third generation photoswitch that primarily targets bipolar cell in retinas undergoing degeneration^6^. DAD has increased solubility compared to second generation photoswitch compounds such as DENAQ and BENAQ, which allows for good tissue penetration *in vitro* as well as *in vivo*. We have previously demonstrated that DAD can be reversibly switched using blue or white light, and it restores light-dependent ganglion cell firing and behavioral responses to light in blind mice (Fig. 2A)^6^. As shown previously, DAD exerts its effects on bipolar cells rather than on RGCs (Fig. 2A–D), which is confirmed by application of blockers for either the excitatory retinal pathway (DNQX and D-AP5) or all synaptic transmission onto RGCs (CdCl_2_) (graphic representation Fig. 2E). We have also demonstrated that application of DAD leads to significant reduction in spontaneous activity in retinal ganglion cells in blind retina, thereby increasing signal to noise ratio of restored light responses. We were interested whether this silencing effect on background firing rate is mediated by reduction of LFPs in general^6^.

To investigate whether photoswitches are able to influence LFP in retina undergoing degeneration, we measured the power spectrum of field potentials using multielectrode array recording and analyzed oscillatory activity from 0 to 40 Hz using the Chronux Matlab Package to calculate the multitaper spectrum over a moving window^20,21^. In wildtype retinas in darkness, only very weak, low frequency oscillatory activity was observed (Fig. S4). A similar low frequency and amplitude oscillatory activity was seen in retinas lacking the light sensitive protein melanopsin in the intrinsically photosensitive retinal ganglion cells (Fig. S4). LFPs were also nearly absent in a model of stationary night blindness, in which the photoreceptor cells are morphologically intact, but lack light-sensing function (*Gnat1*^−/−^, *Gnat2*^−/−^, *Opn4*^−/−^) (Fig. S4)^22–24^. In retina undergoing photoreceptor degeneration, however, strong local field potentials at 5 and 10 Hz are detected (Fig. 2E *before application of photoswitch*). Taken together, these results suggest LFPs are specific to models featuring outer retinal degeneration.

When DAD was applied to blind, degenerated *rd1/rd1*,*Opn4*^−/−^ retina in darkness, LFPs were significantly reduced to frequencies lower than 10 Hz (Fig. S5A,B), which was then similar to wildtype retinas. Additional application of MFA only led to a minor additional decrease in frequency (Fig. S5C–E), but significantly attenuated the light response of DAD itself (Fig. S5D–F). This result was expected as we previously demonstrated that DAD requires the active bipolar-amacrine cell network to function^6^. Interestingly, however, LFPs were nearly completely suppressed when light is switched on in the presence of DAD (Fig. 2E,F).

To establish that this effect is not strain-specific, we also measured local field potential rhythms in the *rd1/rd1*,*Opn4*^−/−^ mouse on a different genetic background (C57Bl6JRj) (Fig. S6). In this mouse line the same effects of reduced LFPs in darkness and elimination of LFP in light were observed.

Taken together, these results support the finding that DAD reduces the overall spiking activity by reduction of LFPs in retinas undergoing degeneration and that DAD exerts its effects in the upstream retinal circuitry, where the oscillations arise.

### DAQ: a charged version of DAD acts on retinal ganglion cells and has no effect on LFP frequency

**Figure 3:**
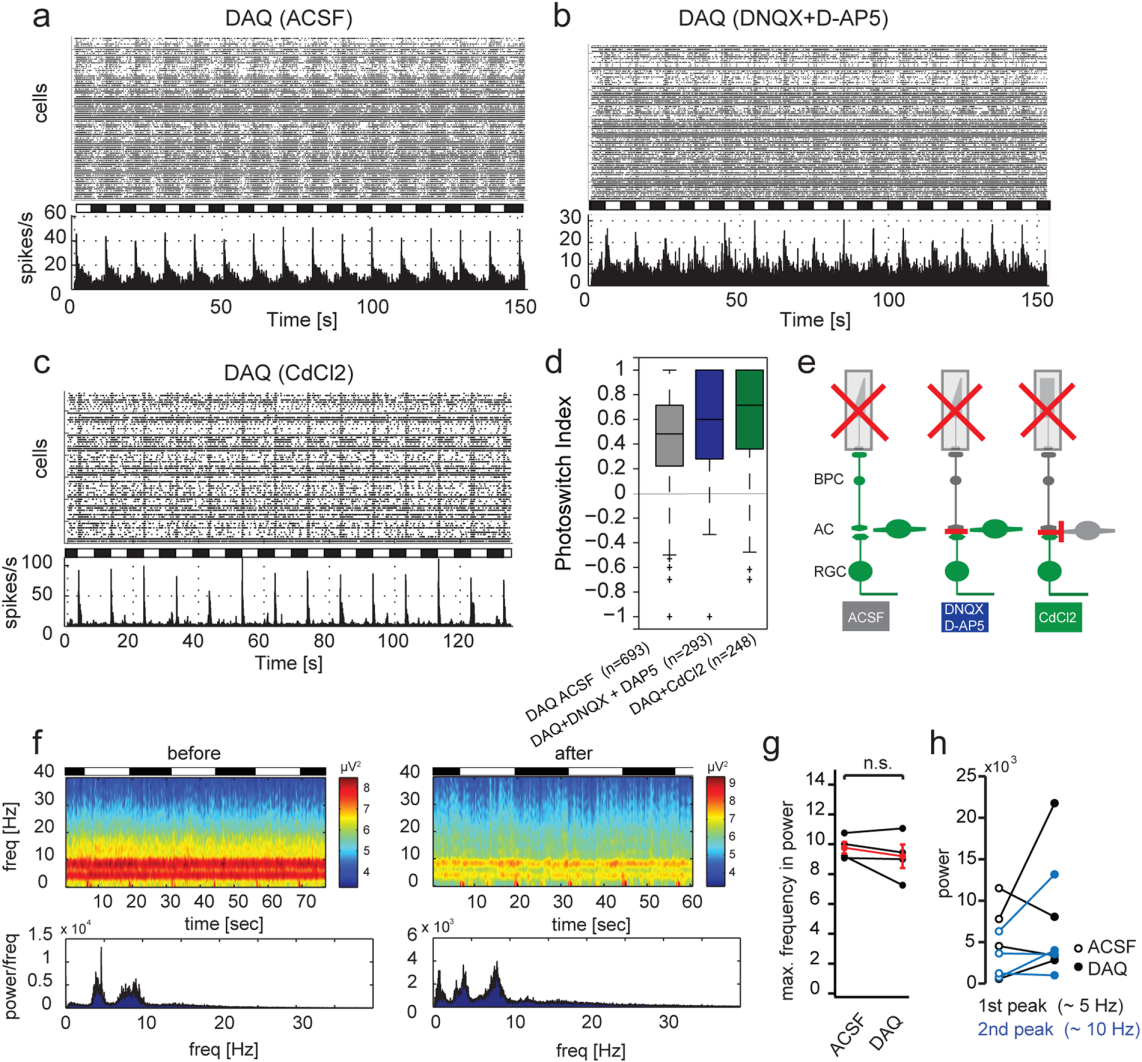
DAQ primarily activates retinal ganglion cells and has minimal effect on LFPs. (**a**) Raster plot and histogram of representative MEA recording in an *rd1/rd1*,*Opn4*^−/−^ retina after treatment with 200 µM DAQ. (**b**) Same as in a, with additional application of DNQX and D-AP5. (**c**) Same as in a, with additional application of CdCl_2_. (**d**) Quantification of light-on responses using the photoswitch index. (**e**) Graphical overview of pharmacological experiments. (**f**) Heat map of rhythmic RGC firing power spectrum before and after application of DAQ. (**g**) Quantification of maximal frequency response before and after application of DAQ (average intensities of complete time range). Maximum frequency with significant power is defined by the peak of the highest frequency reaching a threshold of 4 SD. Each data point refers to the power average of local field potentials in one retina (n = 4 retinas). The average change in power is depicted in red with error bars (mean ± SEM). (**h**) Quantification of maximum power at frequency peaks around 5 (black) and 10 Hz (blue) before and after application of DAQ (empty and filled circles, respectively).

DAQ (Diethylamino-Azo-Quaternary ammonium, Fig. 1B) is a permanently charged version of DAD. After initial characterization of DAQ in layer 2/3 cortical neurons (Fig. S2), we sought to determine DAQ’s properties in blind retina of the *rd1/rd1*,*Opn4*^−/−^ mouse model. We previously demonstrated that the non-permanently charged photoswitch derivative DAD acts primarily on bipolar cells. Although DAD is not permanently charged, the diethyl-groups of DAD are largely protonated in physiological pH (Fig. S7). We therefore expected similar properties of DAQ in blind retina, since DAQ resembles the charged version of DAD. As with DAD, the primary response was observed when light was switched on, and no activation occurred after switching light off (Figs 2, 3). However, the pharmacological profile of DAQ was markedly different from that of DAD. Unlike with DAD, blocking bipolar-to-retinal ganglion cell input by application of DNQX and D-AP5 did not abolish light-dependent activity in the retina (Fig. 3B–D, Table 1). In addition, application of CdCl_2_ (blocking all synaptic transmission) showed a similar result (i.e. persistent light-dependent spiking), thereby demonstrating that neither bipolar cells nor amacrine cells are the primary target cell type affected by DAQ (Fig. 3C, Table 1). These results were unexpected and are in strong contrast to the previously reported pharmacological characterization of DAD, which shows little activation of RGCs but acts primarily through bipolar cells.

**Table 1:** Summary of photoswitch indices for DAD, DAQ, DAA and PhENAQ in absence and presence of pharmacological blockers. Data is represented as mean ± sem.

Compound + condition	PI (mean ± sem)	n retinas (RGCs)
DAD	0.42 ± 0.04	5 (522)
DNQX + AP5	0.06 ± 0.03	3 (162)
CdCl_2_	−0.03 ± 0.04	3 (172)
DAQ	0.46 ± 0.04	8 (693)
DNQX + D-AP5	0.55 ± 0.07	4 (293)
CdCl_2_	0.62 ± 0.1	3 (248)
DAA	−0.09 ± 0.06	12 (970)
DAA off response	0.29 ± 0.06	12 (970)
DNQX + D-AP5	0.19 ± 0.04	6 (543)
CdCl_2_	0.07 ± 0.14	5 (463)
PhENAQ	0.21 ± 0.02	7 (709)
DNQX + AP5	0.33 ± 0.06	3 (260)
CdCl_2_	0.46 ± 0.06	4 (268)

To confirm our MEA results we also performed patch-clamp experiments in bipolar cells from *rd1/rd1* retinas. DAQ was applied with the extracellular solution and patch recordings were performed with no additional blockers present. Cells were filled with a fluorescent dye for posthoc identification of the patched cell (Fig. S8F). At a holding potential of −54 mV, no light-induced currents could be detected (Fig. S8). When we performed current-voltage relationships on the other hand, a strong sustained light-dependent outward current was observed (Fig. S8C–E). This current, however, is unlikely to be the cause of the light induced firing responses seen in RGCs in MEA recordings, as bipolar cell resting potentials lie below −50 mV in degenerated retina^25^. However, these patch clamp experiments demonstrate that lack of light-induced excitatory currents at membrane resting potential is not due to limited accessibility of the photoswitch within the tissue. Light-dependent outward currents were detectable above holding potential of –20 mV and size of these currents are comparable to currents mediated by DAD (80.86 ± 11.64 pA and 64.96 ± 13.19 pA at 40 mV holding potential, respectively, p = 0.44).

Previous studies using these types of photoswitches indicated that these compounds elicited their effects on cellular excitability by modulating voltage-gated K^+^ channels^6^. To determine whether the observed light-evoked spiking in the presence of DAQ was mediated by the same mechanism, we recorded spike responses from individual RGCs after applying DAQ (control condition) and after blocking voltage-gated K^+^-channels (200 μM TEA-Cl). Consistent with the hypothesized site of action at voltage-gated K^+^-channels, this pharmacological manipulation strongly suppressed light-evoked spike responses in ganglion cells, resulting in a significant decrease in photoswitch index (Fig. S9A,B). To gain further insight into the circuit mechanisms mediating the observed light responses, we performed whole-cell, voltage-clamp recordings from individual RGCs following DAQ application. Excitatory and inhibitory synaptic inputs to the cells were isolated by holding a cell’s membrane voltage at the reversal potential for inhibition (−70 mV) or excitation (0 mV), respectively. Light-evoked excitatory synaptic currents were small (<20 pA) and changes in inhibitory synaptic currents were undetectable relative to the leak currents (Fig. S9C). Further, blocking excitatory synaptic input with ionotropic glutamate receptor antagonists (25 μM NBQX; 50 μM D-AP5) slightly, but insignificantly, suppressed light-evoked spike responses in ganglion cells and did not significantly affect the measures photoswitch index relative to control (Fig. S9D,E). Together, these data are consistent with a relatively weak effect of DAQ at the level of retinal bipolar cells, suggesting that the principal site of action within the retinal circuit occurs at the level of the ganglion cells. Furthermore, the results from these patch experiments are consistent with the outcome of the pharmacological experiments on MEA.

Taken together, these results demonstrate that DAQ has a different pharmacological and cell-specificity profile compared to DAD, likely attributable to the permanent charge in the molecule. In this regard, DAQ seems more similar to DENAQ, a second-generation photoswitch. However, DAQ has a much higher solubility in water and buffer than DENAQ (up to 200 mM in H_2_O, comparable to DAD). We therefore consider DAQ as an intermediate between second (DENAQ, BENAQ, PhENAQ) and the third generation photoswitch (DAD).

We next wanted to investigate whether DAQ reduces LFPs in absence or presence of light. When washed onto the retina in the dark or switching between light and dark, DAQ had no effect on LFP frequencies (Fig. 3F,G) and overall power (Fig. 3F,H). This result can most likely be attributed to DAQ targeting retinal ganglion cells and not influencing the upstream circuitry, which is responsible for generating the oscillatory activity.

### DAA: A truncated version of DAD acts upstream of retinal ganglion cells and suppresses rhythmic LFPs

**Figure 4:**
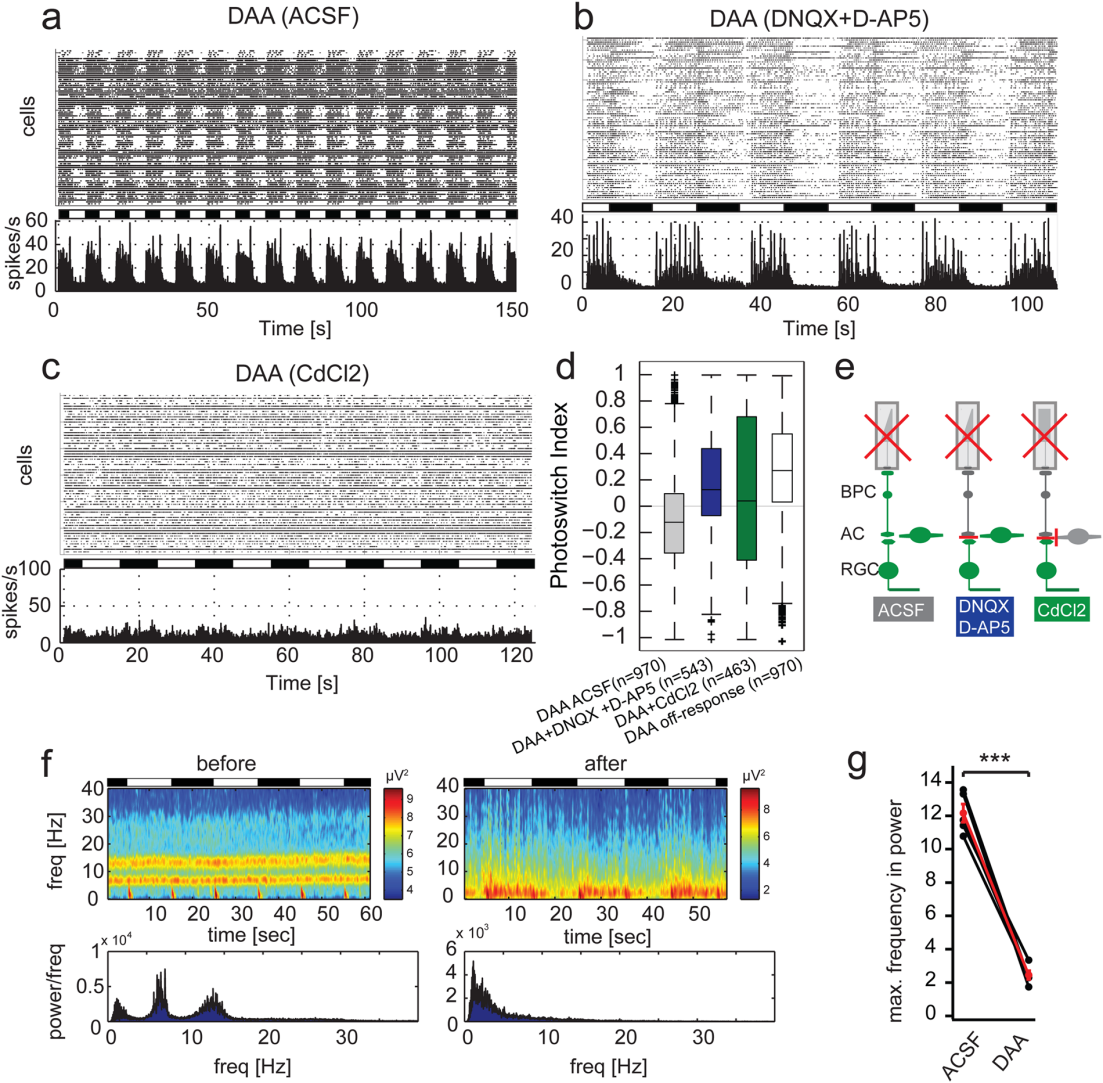
Pharmacological profile of DAA and effect on LFPs in blind retinae. (**a**) Raster plot and histogram of representative MEA recording in an *rd1/rd1*,*Opn4*^−/−^ retina after treatment with 200 µM DAA. (**b**) Same as in a, with additional application of DNQX and D-AP5. (**c**) Same as in a, with additional application of CdCl_2_. (**d**) Quantification of light-on responses using the photoswitch index. (**e**) Graphical overview of pharmacological experiments. (**f**) Heat map of power spectrum of local field potentials before and after application of DAA. (**g**) Quantification of maximal frequency response before and after application of DAA. Maximum frequency with significant power is defined by the peak of the highest frequency reaching a threshold of 4 SD. Each data point refers to the power average of local field potentials in one retina (n = 5 retinas). The average change in power is depicted in red with error bars (mean ± SEM).

Although DAA and DAQ showed relatively similar properties in layer 2/3 neurons of murine brain slice preparations, DAA had strikingly different functionality when applied to blind mouse retina (Fig. 4A–D and Table 1). Unlike all other photoswitches studied to date, the strongest firing was observed when light was switched off (sustained off-response), though a transient light-on response was also detected (Fig. 4D). Because of this unique light response, we next set out to investigate DAA’s action on bipolar and amacrine cells selectively. First, we performed pharmacological experiments blocking bipolar cell input onto retinal ganglion cells using DNQX and D-AP5. Interestingly, application of these blockers led to a full inversion in light response, with emergence of a sustained light-on response. The main spiking activity in presence of these blockers was detected during the light phase and only a transient response was seen when light was switched off (Fig. 4B). This inversion of the photo-response under glutamergic blockage strongly suggests a dominant action of DAA on amacrine cells, analogously to the first-generation photoswitch AAQ^4^. Conversely, application of CdCl_2_ nearly completely abolished light-dependent firing (Fig. 4C,D). These results demonstrate that DAA targets primarily amacrine cells and partially bipolar cells, and that it has only a minor effect on RGCs. It is notable that both uncharged photoswitch compounds (DAA and DAD) have primary effect upstream of retinal ganglion cells while all fast-relaxing charged compounds to date (DAQ, DENAQ, and BENAQ) appear to primarily target retinal ganglion cells directly.

To further substantiate the finding that photoswitches acting upstream from retinal ganglion cells, at the site of LFP origin, reduce the oscillatory activity, we investigated the effect of DAA on rhythmic LFPs (Fig. 4E,F). Similar to DAD, DAA reduced LFPs, with an even stronger reduction than induced by DAD application (Fig. 2E,F). This may be explained by DAA largely targeting amacrine cells, the primary source of the LFPs in the degenerating retina, whereas DAD exerts its effects primarily on bipolar cells.

### PhENAQ: A second generation photoswitch targets retinal ganglion cells and has no effect on LFPs

**Figure 5:**
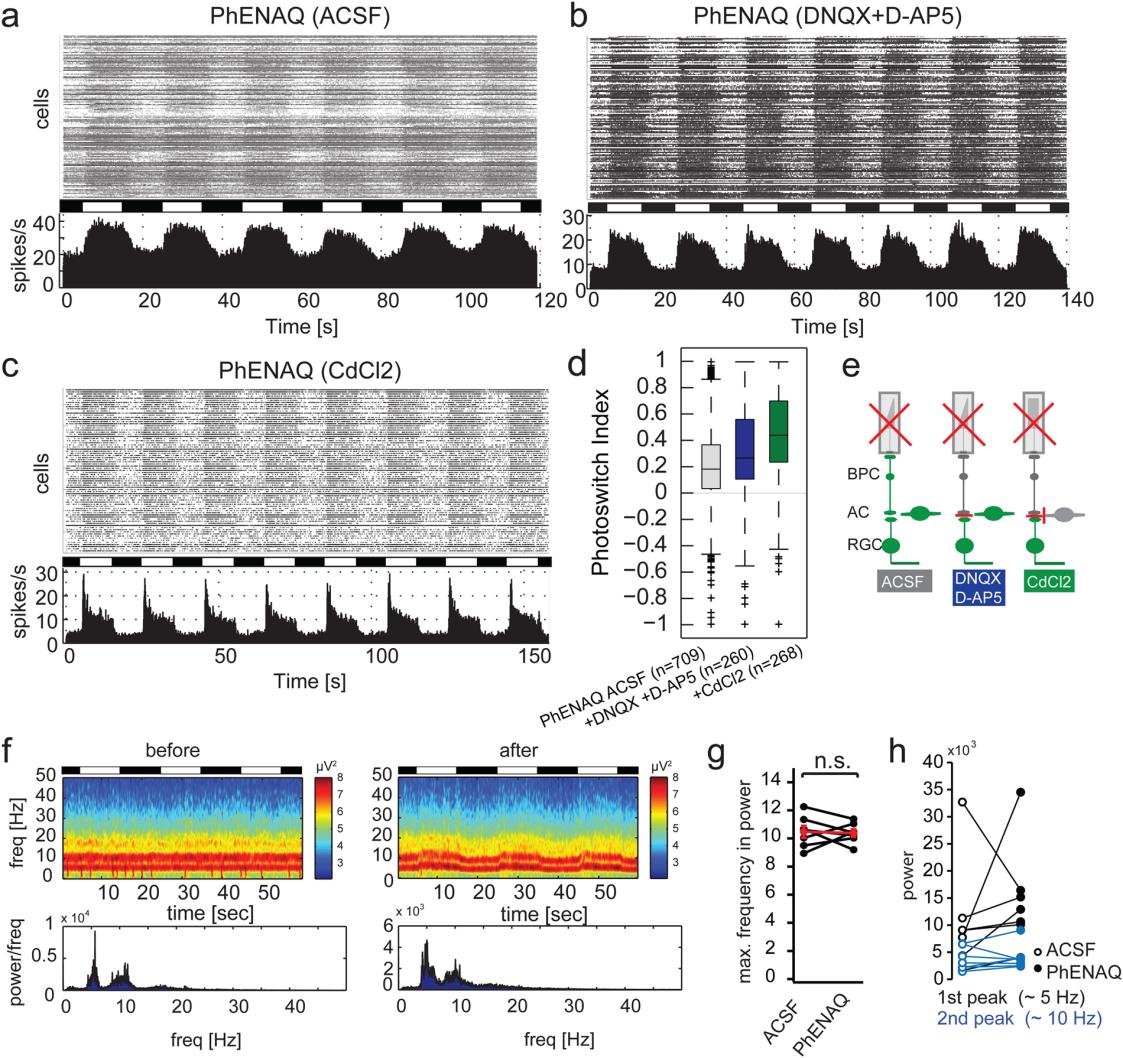
Pharmacological profile of PhENAQ and effect on rhythmic LFPs in blind retinae. (**a**) Raster plot and histogram of representative MEA recording in an *rd1/rd1*,*Opn4*^−/−^ retina after treatment with 200 µM PhENAQ. (**b**) Same as in a, with additional application of DNQX and D-AP5. (**c**) Same as in a, with additional application of CdCl_2_. (**d**) Quantification of light-on responses using the photoswitch index. (**e**) Graphical overview of pharmacological experiments. (**f**) Power spectrum of local field potentials before and after application of PhENAQ. (**g**) Quantification of maximal frequency response before and after application of PhENAQ (average intensities of complete time range). Maximum frequency with significant power is defined by the peak of the highest frequency reaching a threshold of 4 SD. Each data point refers to the power average of local field potentials in one retina (n = 7 retinas). The average change in power is depicted in red with error bars (mean ± SEM). (**h**) Quantification of maximum power at frequency peaks around 5 (black) and 10 Hz (blue) before and after application of PhENAQ (empty and filled circles, respectively).

To extend our knowledge further regarding structure-activity relationships and repression of LFPs by application of photochromic blockers, we also analyzed PhENAQ (Fig. 1B). PhENAQ is a ‘second generation’ photoswitch similar to DENAQ and BENAQ with a permanent charge and a large hydrophobic group^18^. As shown previously, PhENAQ has a rather slow and sustained effect on neuronal firing^18^. When applied to *rd1* retinas, PhENAQ showed primarily sustained light on responses (Fig. 5). Application of DNQX and D-AP5 had no effect on light responses; however, spontaneous activity was reduced (Fig. 5B,D). Application of CdCl_2_, on the other hand, led to transient light on responses (Fig. 5C,D) and strongly decreased spontaneous activity, thereby significantly increasing the signal to noise ratio (Table 1, p = 0.0015). These MEA experiments demonstrate that retinal output is primarily dominated by direct light responses in retinal ganglion cells. However, the fact that light responses were significantly improved upon block of all upstream inputs (i.e. application of CdCl_2_) indicates some effect of PhENAQ on amacrine and bipolar cells. Therefore, we conclude that PhENAQ acts in a similar fashion as the other second generation photoswitches DENAQ and BENAQ (Fig. 1B)^3,26^.

We further investigated the action of PhENAQ on LFPs. As expected for a photoswitch primarily targeting RGCs, application of PhENAQ in darkness had no effect on LFPs (Fig. 5E,F). However, when light stimulation was applied, a mild modulating effect on LFPs became visible (Fig. 5E), consistent with PhENAQ acting on both RGCs and to a minor extent on cells upstream of RGCs. However, no effect on maximal frequency response or overall power was detected (Fig. 5E,H).

## Discussion

Although the pharmacological profiles of DAQ, DAD and DAA in brain slice are very similar, there are marked differences in their cell-type selectivity in retinal tissue. The mechanism of uptake into RGCs has been studied by Tochitsky *et al*. for various second generation open-channel blockers, such as DENAQ^5^. In contrast to second-generation compounds, membrane-permeable, photoswitchable open-channel blockers appear to target different cell types (bipolar for DAD and amacrine cells for DAA) and do not appear to rely on an uptake through dilated channels. Although Tochitsky *et al*. have demonstrated how second generation open-channel blockers enter into retinal ganglion cells of degenerated retinas (via P2X receptors), the important question of what prevents the supposedly membrane-permeable photoswitches such as DAD and DAA from diffusing through all cell types’ membranes, remains to be answered^6^.

Rhythmic LFPs arising in blind retinas are an obstacle for vision restoration approaches, as the oscillatory activity increases the general background activity and therefore decreases the signal-to-noise ratio for restored signals^16^. Interestingly, in wild-type retina, in a melanopsin knock-out model and in a model for stationary night-blindness, in which rod and cones are not light-responsive, rhythmic LFPs around 5 and 10 Hz were not detected. In contrast, in two studied retinal degenerate mouse models (*rd1* and *rd10*), these characteristic LFPs were present, even at an early stage of the degenerative process^13^. These results indicate that although not fully functional, the presence of morphologically intact photoreceptor cells can prevent the induction of LFPs and that LFPs are primarily related to the degeneration of photoreceptor cells. However, after partial bleaching of healthy retina, which leaves photoreceptor cells intact, some electrophysiological features of LFPs comparable to rd10 mice can be mimicked^27^.

The majority of the pathological hyperactivity in degenerated retinas is thought to arise from AII amacrine – bipolar cell coupling and from a second network in the outer retina, which has been recently described by Euler and Schubert^28^. These multiple sources of LFPs may complicate the elucidation and understanding of a possible mode of action of pharmaceuticals to target them^28–30^. Pharmacological intervention by blocking gap junctions eliminates most of the aberrant activity and improves optogenetically-evoked light responses^16,31^. While blocking gap junction coupling between AII amacrine and bipolar cells might be beneficial for approaches targeting RGCs directly, it will not be applicable for strategies targeting the retinal network upstream from RGCs. However, restoring vision in the upper retinal network is able to induce light on and off responses, which might be advantageous for the perceived spatial and temporal resolution in blind patients^6,32,33^.

We have now investigated the effect of different classes of photoswitches on these LFPs. MEA recordings of degenerated retina with photoswitches were filtered for low frequencies. In combination with pharmacological evaluation of the photoswitches, we conclude that molecules that exert their effect via RGCs (i.e. PhENAQ and DAQ) have no effect on the magnitude and frequency of LFPs, while photoswitches that act on cell types upstream of RGCs can be used to suppress LFPs. Furthermore, DAA, which primarily exerts its effect on amacrine cells, reduces LFPs to a greater extent than DAD, which primarily acts on bipolar cells. This is further supported by the fact, that light stimulation has opposing effects on LPF modulation, i.e. light stimulation depresses LFP further in presence of DAD but increases with DAA (Figs 2f, 4f, respectively). This data is in line with the current understanding that LFPs arise from ON bipolar cells and AII amacrine cells, which is further supported by the finding that blockage of glutamatergic input onto RGCs decreases local field potentials, however, fails to abolish the high spontaneous firing rates^13,15,25,29^.

PhENAQ, a photoswitch with strong actions on RGCs, fails to decrease overall LFPs, however, light dependent modulation is observed. In addition, the pharmacological experiments also indicate light-dependent activation in amacrine and bipolar cells (Fig. 5). This lack of overall LFP reduction could partially be explained, if bipolar and amacrine cell responses cancel each other out. To investigate the exact specificity and functionality of PhENAQ further studies need to be performed.

To determine if these conclusions are generalizable to all outer retinal degeneration models further testing in other retinal degenerate models will be required. In addition, it remains to be seen whether optogenetic stimulation of the upstream retinal circuitry or photochromic ligands targeting synaptic receptors rather than ion channels are also able to suppress local field potentials^34^. Furthermore, photoswitches targeting cells upstream from RGCs induce slight modulations in LFP generation depending on light application. These modulations are probably due to different binding affinities of the photoswitches in light and darkness; whereby incomplete depression of LFPs is induced in one state and not the other. It remains to be seen whether these fluctuations may present further challenges to the visual processing in higher brain areas.

Chemical modification of azobenzene-derivatives of QX-314 and lidocaine demonstrate the remarkable sensitivity of the retina to small structural changes of these compounds. Different derivatives appear to target different cell types, with DAD targeting bipolar cells primarily, DAA targeting amacrine cells, and DAQ primarily targeting retinal ganglion cells. The retinal ganglion cell outputs of blind retinas treated with these compounds are distinct, with DAD-treated retinas showing strong transient on- and off- responses, DAA retinas showing a sustained off-response to alternating light-dark stimuli, and DAQ showing responses similar to second generation compounds PhENAQ and DENAQ, with primarily sustained-on responses. However, even within a group targeting the same cell class, compounds may show distinctly different physical properties, such as PhENAQ and DAQ, with DAQ being much more soluble than PhENAQ. The substantial variety of responses and physical properties of closely-related photoswitch derivatives of QX-314 and lidocaine suggest that additional medicinal chemistry may result in compounds further optimized for use in vision restoration in outer retinal degeneration.

Our results confirm that photochromic ligands are a potentially powerful pharmacological approach for the restoration of vision lost to outer retinal degeneration. The strong suppression of rhythmic local potentials by DAA and DAD indicate that members of this class may be particularly useful for vision restoration by providing higher signal-to-noise output as well as potentially recapitulating retinal processing by circuitry upstream of the ganglion cell.

## Methods

### Chemicals

Photoswitch derivatives were synthesized as HCl salts in accordance with the synthetic routes described in the *Supplementary Methods*. All other chemicals were purchased from Abcam or Tocris Bioscience.

### Animals and retina and acute brain slice preparation

For all MEA and retina patch clamp recordings we used 3- to 7-month-old homozygous *rd1/rd1 Opn4*^−/−^ mice (C3H/HeJ strain, Charles River Laboratories), if not otherwise indicated. Furthermore, we investigated local field potentials in *rd1/rd1 Opn4*^−/−^ mice (C57Bl6JRj background), in wild type (C57Bl6JRj) and *Opn4*^−/−^ mice [gift of Satchin Panda, Salk Institute, La Jolla, California, USA]) animals as well as in the blind, but nondegenerating mouse line (*Gnat1*^−/−^ *Gnat2*^−/−^ *Opn4*^−/−^, derived from Gnat1^tm1Clma^ and B6.Cg-Gnat2^cpfl3/Boc^, The Jackson Laboratory). Mice were sacrificed by cervical dislocation. Retinas were dissected and kept in artificial cerebrospinal fluid (ACSF) at room temperature containing 125 mM NaCl, 2.5 mM KCl, 1.25 mM NaH_2_PO_4_, 1 mM MgCl_2_, 2 mM CaCl_2_, 26 mM NaHCO_3_, and 20 mM D-glucose; aerated with 95% O_2_/5% CO_2_.

All animal use procedures were approved by the University of Washington Institutional Animal Care and Use Committees. All experiments were performed in accordance with relevant guidelines and regulations.

### Coronal slice preparation

For basic characterization of photoswitches acute coronal brain slices were prepared from C57Bl6JRj mice (postnatal day 10-13, both male and female animals were used without known experimenter bias). Mice were decapitated and the brain was rapidly removed and kept in ice-cold saline solution composed of (in mM): 87 NaCl, 2.5 KCl, 1.25 NaH_2_PO_4_, 25 NaHCO_3_, 0.5 CaCl_2_, 7 MgCl_2_, 25 glucose, 75 sucrose saturated with carbogen (95% O_2_/5% CO_2_). Coronal brain slices (250 μm) were prepared using a Campden vibratome 7000 smz^2^ (NPI Electronic). Slices were incubated at 34 °C for 30 min in ACSF and further stored at room temperature from 1 to 5 h before recordings were started.

### Retina slice preparation

Retinal slice preparations were performed as previously described in^6^. Briefly, retinas were embedded in 3% low-melting agarose and cut into a small block. The agar block with the retina was glued to the specimen disc. A Campden vibratome 7000 smz-2 (NPI Electronic) was used to cut slices with a thickness of 400–450 μm, which were directly transferred to the recording chamber. A slice anchor was mounted to hold down the agar, while the retina was unobstructed.

### Patch-Clamp recordings of layer 2/3 cortical neurons

All experiments were performed at room temperature and in presence of 1 μM TTX unless stated otherwise. Photoswitch solutions were prepared in ACSF was prepared from a 200 mM stock solutions (ddH_2_O), except for DENAQ, which was prepared in DMSO. Holding potential was set to −60 mV. To evaluate blocking/unblocking K_v_ channels kinetics of photoswitches, cells were depolarized from a holding potential of −60 mV to +50 mV. During the depolarization, pulse the photoswitch was switched from TRANS (dark) to CIS (460 nm) and back. Light-induced currents were corrected for desensitization, and T was calculated from this photoswitch-mediated current trace. For investigation of photoswitch-mediated effects on Na_v_-channels, a voltage jump from −70 mV to −40 mV was performed in the absence of TTX, but in presence of TEA and cesium. Cells were patched using glass electrodes with a resistance of 6−7 MΩ and an intracellular solution containing (in mM): 140 K-gluconate, 10 HEPES, 12 KCl, 4 NaCl, 4 Mg-ATP, 0.4 Na_2_-GTP. Recordings were made with an EPC 10 USB amplifier, which was controlled by the Patchmaster software (HEKA). Data was filtered at 2.9−10 kHz and digitized at 50 kHz. Cells with leak currents were >200 pA for hippocampal neurons or with a series resistance >15 MΩ were rejected from analysis. Data was analyzed using the Patcher’s Power Tools (MPI Göttingen) and routines written in IgorPro (Wavemetrics).

### Patch-clamp recordings in whole mount retinas

Retinal Ganglion Cells: Patch-clamp recordings were performed using borosilicate glass pipettes containing Ames medium for extracellular spike recording or, for whole-cell recording, a cesium-based internal solution containing: 105 mM Cs-methansulfonate, 10 mM TEA-Cl, 20 mM HEPES, 10 mM EGTA, 2 mM QX-314, 5 mM Mg-ATP, and 0.5 mM Tris-GTP, pH ~7.3 with CsOH, ~280 mOsm. Series resistance (~3–9 MΩ) was compensated online by 50%. Excitatory and inhibitory synaptic currents were isolated by holding cells at the reversal potentials for inhibition/chloride (−70 mV) and excitation (0 mV), respectively. Data were acquired at 10 kHz using a Multiclamp 700B amplifier (Molecular Devices), Bessel filtered at 3 kHz, digitized using an ITC-18 analog-digital board (HEKA Instruments), and acquired using the Symphony acquisition software package (http://symphony-das.github.io).

### Patch-clamp recordings in retinal slices

Retinal slices were transferred to the recording chamber immediately after slicing. Electrodes with a resistance of 12–14 MΩ and with an intracellular solution containing either 140 mM K-gluconate, 10 mM HEPES, 12 mM KCl, 4 mM NaCl, 4 mM Mg-ATP, 0.4 mM Na-GTP, and1% Lucifer Yellow, pH 7.3, with KOH or 120 mM Cs-methansulfonate, 5 mM TEA-Cl, 10 mM HEPES, 3 mM NaCl, 10 mM EGTA, 2 mM QX-314, 2 mM Mg-ATG, 0.3 mM Na-GTP, and 1% Lucifer Yellow were used for patch clamp recordings in retinal bipolar cells. Bipolar cells were patched with the same amplifier and settings used as for cortical slice patch clamp recordings. Bipolar cell recordings with >50 pA and >25 MΩ were excluded from analysis.

### Multielectrode Array (MEA) electrophysiology

For extracellular recordings, a whole-mount retina was placed onto a multielectrode-array (MEA 1060-inv-BC, Multi-Channel Systems) with the retinal ganglion cell layer down. We used 8 × 8 rectangular arranged MEA electrodes with a diameter of 30 μm and a spacing of 200 μm (200/30 ITO, Multichannel Systems). After mounting on the MEA, retinas were washed for at least 20 min and recordings were made at 34 °C, while constantly perfusing ACSF without photoswitch. Retinas were then perfused with 200 μM photoswitch solution for up to 10 minutes and afterwards again with ACSF. Recordings of spiking as well as LFP recordings were continued for up to 4 hours post photoswitch application. Effects on firing rate and LFPs were constant during this time. Extracellular spikes were high-pass filtered at 300 Hz and digitized at 20 kHz. A spike threshold of 4 SD (standard deviation) was set for each channel. Typically electrodes with a width of 30 μm record from one to three RGCs. Analysis of spike waveforms was performed using Plexon Offline Sorter (version 3) analyzing principle component analysis of spike waveforms. Blocking excitatory input on retinal ganglion cells is achieved by perfusion of 25 μM DNQX and 50 μM D-AP5. In order to completely block all synaptic transmission in the retina and isolate retinal ganglion cell responses 500 μM CdCl_2_ was applied. Blockade of gap junctions in the retina was achieved by perfusion of meclofenamic acid (MFA).

### Light stimulation

MEA recordings were performed using a xenon light source (Sutter Instruments) through a liquid light guide and diffusing filter (Thorlabs Inc.). Light stimuli were delivered and monitored by a computer-controlled shutter (Vincent Associates). In patch clamp recordings light was applied using a Polychrome V (Till Photonics) controlled through the Patchmaster software.

### Data analysis

RGC firing rate was calculated in 100 ms bins for individual retinas in light and in darkness. The Photoswitch Index was calculated in order to normalize light-elicited changes in firing rate of individual retinal ganglion cells and plotted as interquartile range (Boxplot). PI = (firing rate in the light − firing rate in darkness)/(firing rate in the light + firing rate in darkness). Data analysis was performed using custom routines in IgorPro software (Wavemetrics) or Matlab. In order to analyze oscillatory activity between 0 to 40 Hz we used the Chronux Matlab Package to calculate the multitaper spectrum over a moving window (mtspecgramc)^20,21^. Raw data was converted to hdf5-files with no further preprocessing. Settings parameters were set to (‘tapers’, [5 9], ‘Fs’, 40000, ‘fpass’, [1 40], ‘pad’, 1, ‘trialave’, 1). Power is represented as µV^2^.

For analysis of ‘maximum frequency in power’ the peak of the highest frequency reaching a threshold of 4 SD was measured.

### Statistics

Statistical significance was calculated using the Wilcoxon rank sum test in Matlab. Results were considered significant with *p < 0.05, **p < 0.01, and ***p < 0.001. Error bars are presented as mean ± standard error of the mean (sem).

## Supplementary information

Supplementary Information

## Data Availability

The datasets generated during and/or analyzed during the current study are available from the corresponding author on reasonable request.
