# Supplementary material for: Photopharmacologic Vision Restoration Reduces Pathological Rhythmic Field Potentials in Blind Mouse Retina
Source: Sci Rep. 2019 Sep 19;9:13561. doi: 10.1038/s41598-019-49999-w (PMC6753071; doi:10.1038/s41598-019-49999-w)
Supplement: Supplementary file 1 — Supplementary Information [file 41598_2019_49999_MOESM1_ESM.pdf]

Supplementary Information

Photopharmacologic Vision Restoration Reduces Pathological Rhythmic Field Potentials in Blind Mouse Retina

Katharina Hüll[1,2], Tyler Benster[3,4], Michael B. Manookin[3], Dirk Trauner[2], Russell N. Van Gelder*[3,5] and Laura Laprell*[3,6]

*Corresponding authors

1 Center for Integrated Protein Science Munich and Department of Chemistry, Ludwig-Maximilians-Universität München, Munich, Germany.
2 Department of Chemistry, New York University, 100 Washington Square East, New York, New York 10003-6699, United States
3 Department of Ophthalmology, University of Washington School of Medicine, Seattle, Washington, USA.
4 Present address: Neurosciences Graduate Program, Stanford University, Stanford, CA 94305, USA
5 Departments of Biological Structure and Pathology, University of Washington School of Medicine, Seattle, Washington, USA.
6 Department of Synaptic Physiology, Center for Molecular Neurobiology, ZMNH, University Medical Center Hamburg-Eppendorf, Hamburg, Germany.

Email: laura.laprell@zmnh.uni-hamburg.de, russvg@uw.edu

Table of Contents

Supplementary Figures 2
Supplementary Methods 10
Synthetic Procedures 13
NMR spectra 24
Supplementary References 36

Supplementary Figures

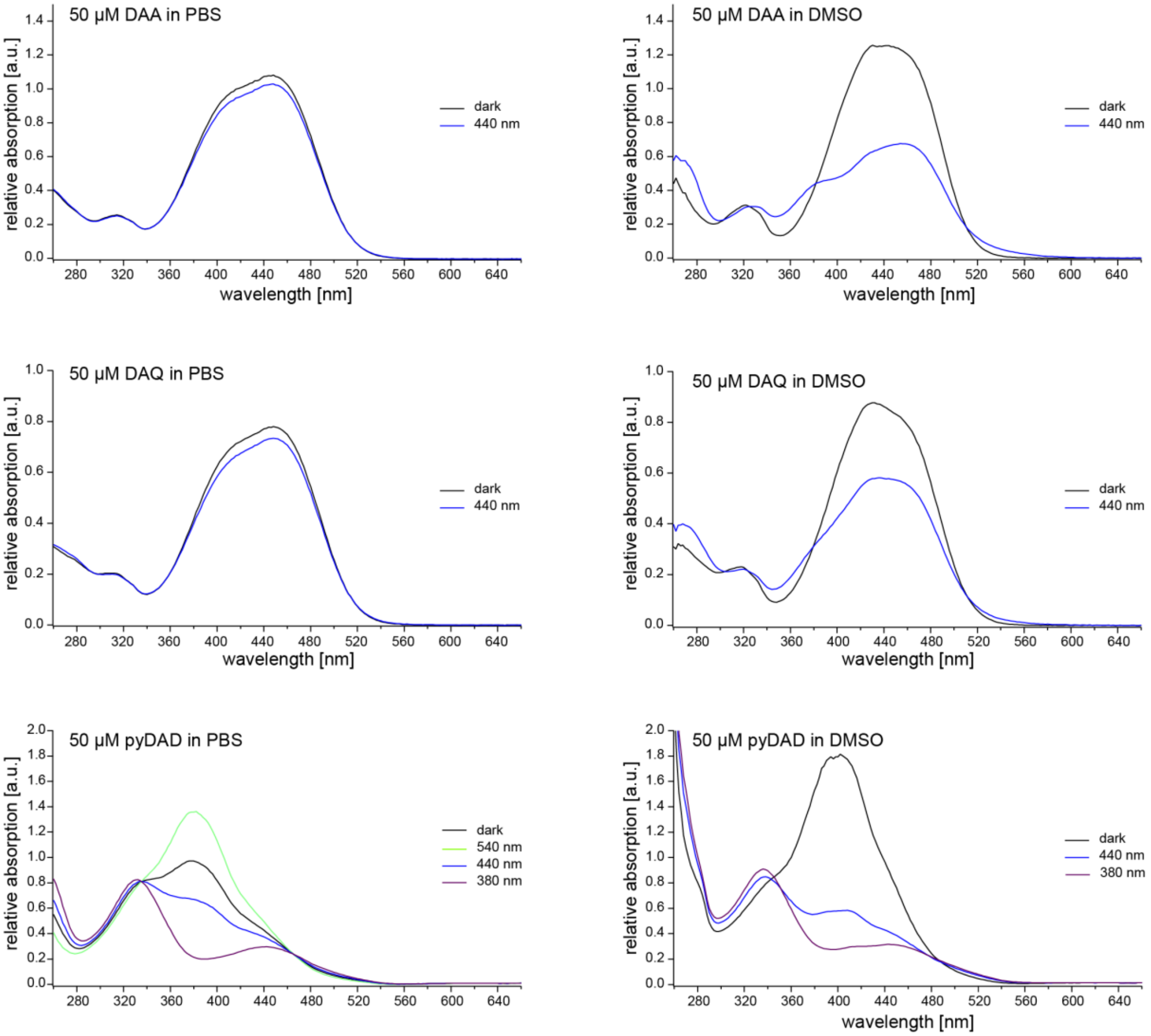

Supplementary Figure 1. UV Vis spectra of DAA, DAQ and pyDAD in PBS and DMSO. Spectra were collected at 50 µM concentration and samples were irradiated for 3 min with monochromatic light of the respective wavelength before collecting the spectrum.

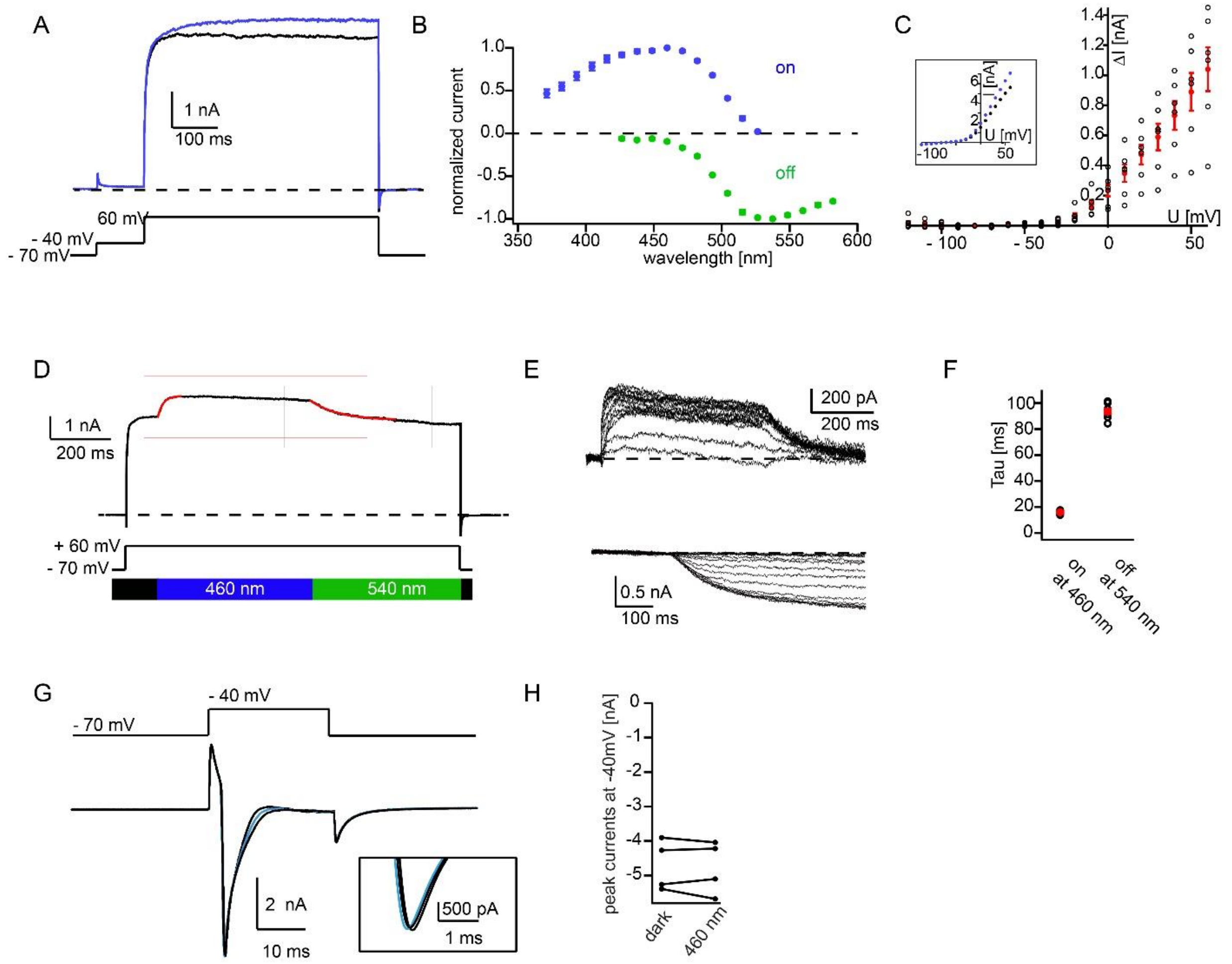

Supplementary Figure 2. **Characterization of DAQ in layer 2/3 cortical neurons of an acute brain slice of wild type mice.** (A) Whole-cell recording after incubation with 200 µM DAQ in the presence of 1 µM TTX. Potassium (K_v) outward currents were activated by a step from –70 mV to +60 mV. Currents in darkness (black) are compared to currents in the presence of 460 nm light. (B) Normalized change in K_v current in DAQ-treated cortical neurons in response to stimulation with light of different wavelengths. (C) Difference in current-voltage relationship between darkness and 460 nm light. Inset: Representative raw data traces in darkness (black) and in 460 nm light (blue). (D) Example trace for determining the kinetics of unblocking the pore of K_v-channels. (E) Top: Raw data currents at +60 mV holding potential, while switching between different wavelengths of light and darkness. Bottom: Raw data traces of decay time by different wavelengths of light (F) Quantification of on- and off-kinetics in response to different wavelengths. $\tau_{unblock}$ = 16.0±0.49 ms (n = 7 cells). Fastest off-responses were achieved at 520 nm light (τ_{block} = 93.51±2.12 ms, n = 7 cells). (G) Recording in cortical neurons in presence of Cesium and TEA. Voltage jump from –70 mV resting potential to –40 mV induces sodium channel currents. (H) Quantification of peak sodium currents in darkness and under blue light illumination.

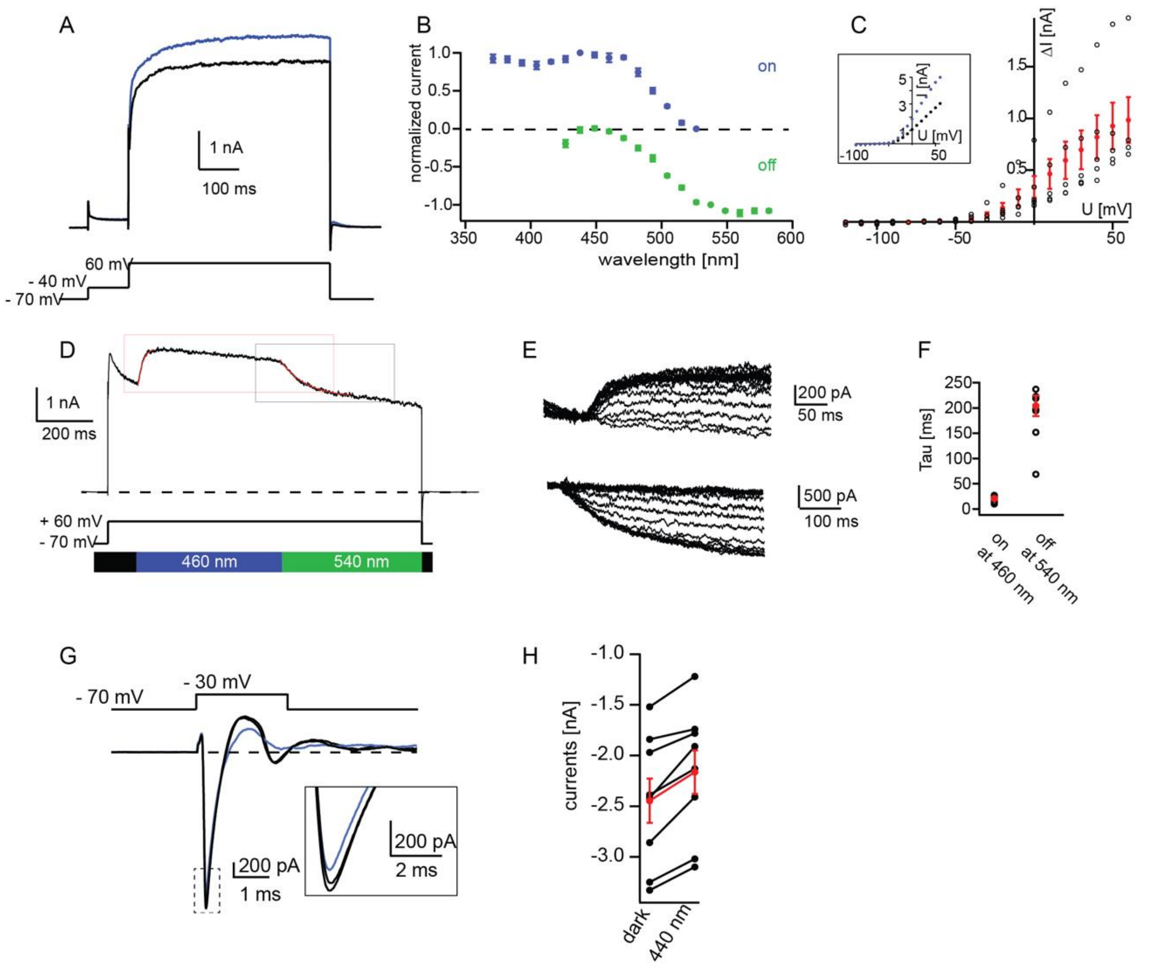

Supplementary Figure 3. **Characterization of DAA in layer 2/3 cortical neurons of an acute brain slice of wild type mice**. (A) Whole-cell recording after incubation with 200 µM DAA in the presence of 1 µM TTX. Potassium (K_v) outward currents were activated by a step from –70 mV to +60 mV. Currents in darkness (black) are compared to currents in the presence of 460 nm light. (B) Normalized change in K_v current in DAA-treated cortical neurons in response to stimulation with light of different wavelengths. (C) Difference in current-voltage relationship between darkness and 460 nm light. Inset: Representative raw data traces in darkness (black) and in 460 nm light (blue). (D) Example trace for determining the kinetics of unblocking the pore of K_v-channels. (E) Top: Raw data currents at +60 mV holding potential, while switching between different wavelengths of light. Bottom: Raw data traces of decay time by different wavelengths of light. (F) Quantification of on- and off-kinetics in response to different wavelength. $\tau_{unblock}$ = 20.4±1.75 ms (n = 9 cells). Fastest off-responses were achieved at 520 nm light (τ_{block} = 204±20.3 ms, n = 9 cells). (G) Recording in cortical neurons in presence of Cesium and TEA. Voltage jump from –70 mV resting potential to 0 mV induces sodium channel currents. (H) Quantification of peak sodium currents in darkness and under blue light illumination.

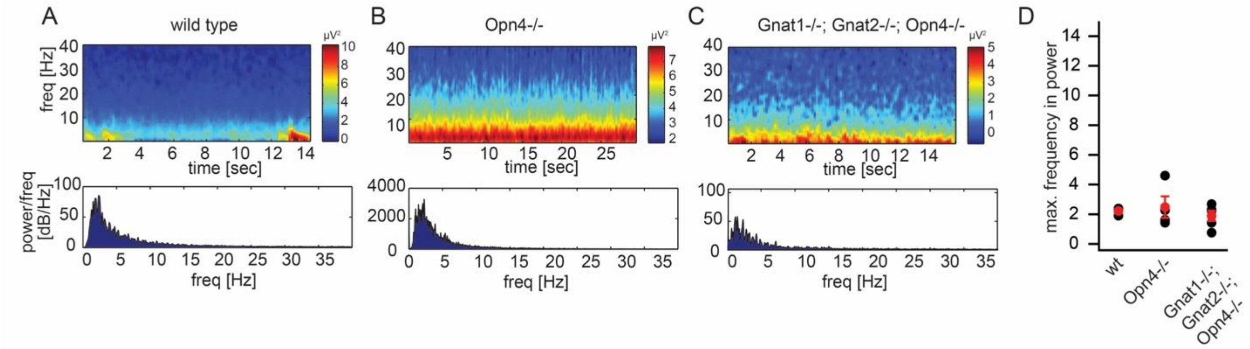

Supplementary Figure 4. Power spectrum of rhythmic LFP in non-degenerating retinas. (A) Local field potentials in wild type animals, (B) *Opn4*$^{-/-}$ and (C) *gnat1*$^{-/-}$*; gnat2*$^{-/-}$, *Opn4*$^{-/-}$ animals. (D) Quantification of maximal frequency response (average intensities of complete time range).

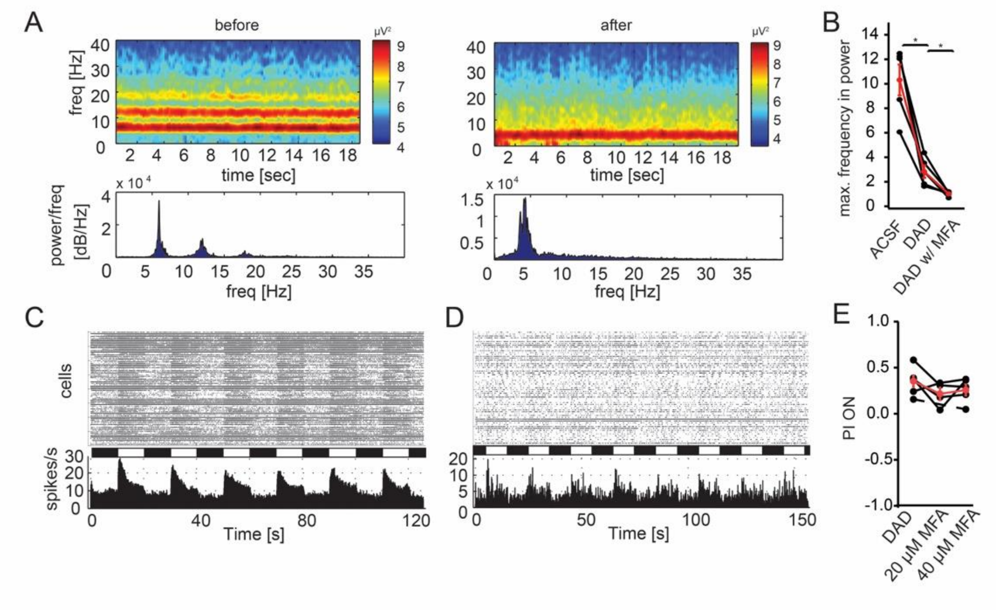

Supplementary Figure 5. DAD represses LFPs in retina undergoing degeneration. (A) Power spectrum of local field potentials before and after application of DAD without light stimulation. (B) Quantification of maximal frequency response before and after application of DAD and after application of meclofenamic acid (MFA) (average intensities of complete time range). (C) Raster plot and histogram of representative MEA recording in an *rd1/rd1; Opn4*$^{-/-}$ retina after treatment with 200 µM DAD. (D) Same as in a, with additional application of MFA. (E) Quantification of light-on responses using the photoswitch index.

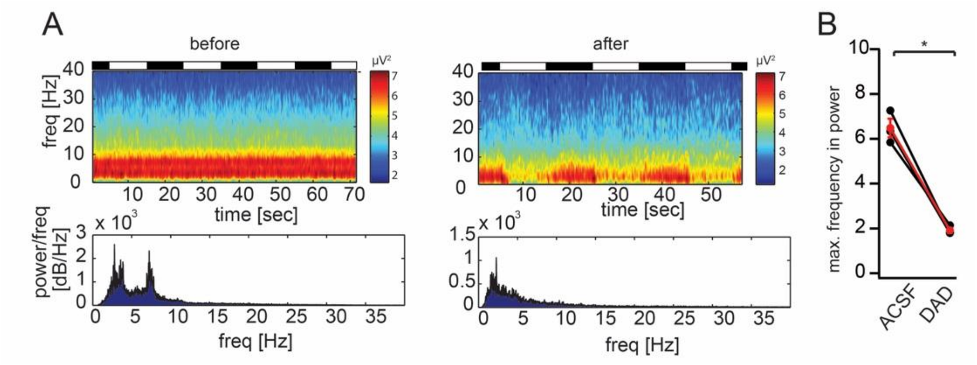

Supplementary Figure 6. DAD represses local field potentials in a *rd1* mouse model with C57Bl/6 genetic background. (A) Power spectrum of oscillatory local field potentials before and after application of DAD. (B) Quantification of maximal frequency response before and after application of DAD. (average intensities of complete time range).

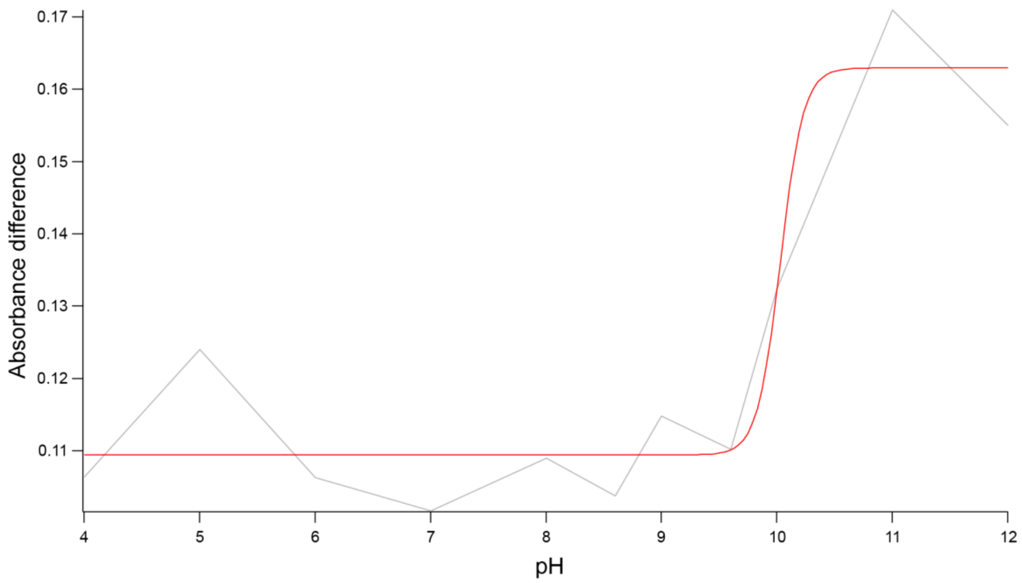

Supplementary Figure 7. pK$_A$ determination of DAD according to a procedure by Ríos Martínez and Dardonville.[1] Sigmoidal fit using Igor 6.37 estimated a pK$_A$ of 10 for DAD. Data was collected as a single measurement.

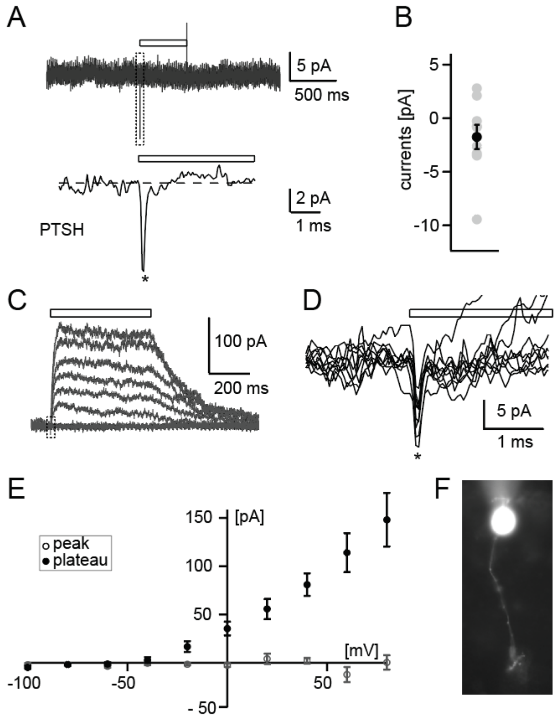

Supplementary Figure 8. **DAQ has no effect in retinal bipolar cells from *rd1/rd1,Opn4*$^{-/-}$ retinas** (A) Voltage-clamp recording of a bipolar cell in presence of DAQ. Top: Full trace, peristimulus time histogram (PTSH) of 5 sweeps. Asterix (*) marks a light artefact induced by the LED. Bottom: Enlargement of dotted box on top. Bar above the trace marks the light stimulation with 460 nm light. (B) Peak-currents measured at 0.5 ms after light onset (average time to peak for DAQ-mediated light responses in bipolar cells [2] (C) Raw data traces of an IV-relationship of DAQ mediated currents in the absence of blockers. (D) Raw data trace for voltages from –100 to +40 mV. Enlargement of box in C. (E) Analysis of IV relationships. Empty circles: Transient peak current. Filled circles: Late K_v-channel component. (n = 8). (F) Fluorescence image of a bipolar cell filled with Lucifer Yellow after whole cell patch clamp configuration. Data is represented as mean±sem.

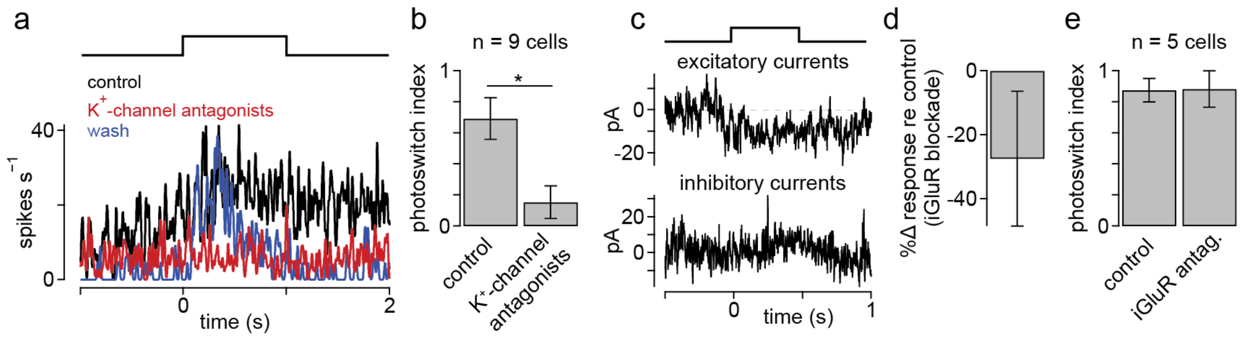

Supplementary Figure 9 K$^+$-channel antagonists attenuate DAQ-mediated light responses. (A) Spike rate from a ganglion cell recorded to a 1-s light flash following DAQ application. Recordings were made under control conditions (black), following the application of K$^+$-channel antagonists (red), and after washing out the drugs (blue). Spike rate was strongly attenuated during the light flash following drug application. (B) The photoswitch index was significantly suppressed following application of K$^+$-channel antagonists (n = 9 cells; p = 2.0 x 10^{-2}). (C) Excitatory and inhibitory synaptic current recordings in the same cell following application of DAQ. Synaptic currents were relatively small. (D) On average, the ionotropic glutamate receptor antagonists suppressed ganglion cell spike responses by ~25%, but this effect was not statistically significant (n = 5 cells; p = 0.16). (E) The photoswitch index was not significantly decreased following application of ionotropic glutamate receptor antagonists that attenuated excitatory synaptic input to the recorded ganglion cells (n = 5 cells; p = 0.25). Bars indicate mean ± SEM. All statistical tests were paired and were performed using the Wilcoxon signed rank test. Control conditions refer to conditions where only the photoswitch DAQ is applied.

Supplementary Methods

Chemical Synthesis

General Experimental Techniques

Unless stated otherwise all reactions were carried out with magnetic stirring using oven-dried glassware (160 °C) under inert gas atmosphere (nitrogen or argon). Syringes used to transfer reagents and solvents were purged with nitrogen prior to use. Low temperature reactions were carried out in a Dewar vessel filled with the appropriate cooling agent e.g. H_2O/ice (0 °C). Heating was conducted using a heated oil bath. Yields refer to spectroscopically pure compounds unless otherwise stated.

Solvents and Reagents

Reaction solvents were purchased from Acros Organics as 'extra dry' over molecular sieves and handled under inert gas atmosphere. Tetrahydrofuran (THF) was distilled from Na/benzophenone prior to use. Dichloromethane (DCM), triethylamine (TEA) and diisopropylethylamine (DIPEA) were distilled from calcium hydride. Ethanol was purchased from commercial suppliers and used as received. Solvents for extraction and flash column chromatography were purchased in technical grade purity and distilled under reduced pressure on a rotary evaporator prior to use. All other reagents and solvents were purchased from commercial suppliers and used as received.

Chromatography

Reactions and chromatography fractions were monitored by qualitative thin-layer chromatography (TLC) on silica gel F254 TLC plates from Merck KGaA. Analytes were visualized by irradiation with UV light and/or by immersion of the TLC plate in ninhydrin or potassium permanganate solution followed by heating with a hot-air gun. Flash column chromatography was performed Geduran® Si60 (40-63 µm) silica gel from Merk KGaA (eluents are given in parenthesis). Reverse Phase column chromatography was performed on Waters C18 (C18; 55-105 µm, 125 Å) as stationary phase (eluents are given in parenthesis). LC-MS was performed on an Agilent 1260 Infinity HPLC System, MS-Agilent 1100 Series, Type: 1946D, Model: SL, equipped with a Agilent Zorbax Eclipse Plus C18 (100 x 4.6 mm, particle size 3.5 micron) reverse phase column with a constant flow-rate of 1 mL/min and a 10 → 100% MeCN/H_2O + 0.1% FA gradient over 10 min.

NMR Spectra

NMR spectra were measured on Varian 400 MHz Bruker AVIII HD (cryoprobe) for proton nuclei (100 MHz for carbon nuclei respectively). The ^1H NMR shifts are reported in parts per million (ppm) related to the chemical shift of tetramethylsilane. ^1H and ^{13}C NMR shifts were calibrated to the residual solvent signals: $CDCl_3$ (7.26 ppm/77.16 ppm) and DMSO (2.50 ppm/39.5 ppm). ^1H NMR spectroscopic data are reported as follows: Chemical shift in ppm (multiplicity, coupling constants (Hz), integration). The multiplicities are abbreviated as follows: s (singlet), d (doublet), t (triplet), q (quartet) and m (multiplet) and are reported as observed. Except for multiplets, the chemical shift of all signals is reported as the centre of the resonance range. Additionally, to ^1H and ^{13}C NMR measurements, 2D NMR techniques as homonuclear correlation spectroscopy (COSY), heteronuclear single quantum coherence (HSQC) and heteronuclear multiple bond

coherence (HMBC) were used to assist signal assignment. All raw fid files were processed, and the spectra analyzed using the program MestReNova from Mestrelab Research S. L.

Mass Spectra

All high-resolution mass spectra (HRMS) were recorded by the LMU Mass Spectrometry Service. HRMS were recorded on a MAT 90 from Thermo Finnigan GmbH using electrospray ionisation (ESI) or a MAT 90 from Jeol Ltd. using electron ionization (EI).

UV/Vis Spectra

UV/Vis spectra were recorded on a Varian Cary 50 Scan UV/Vis spectrometer using Helma SUPRASIL precision cuvettes (10 mm light path). All compound stock solutions were prepared under benchtop light conditions at 50 mM in DMSO and diluted to the right concentration in the final solvent (DMSO or PBS). Photoswitching was achieved using a Polychrome V (Till Photonics) Monochromator, or a Prizmatix ultra high power LED (460 nm), connected to a fiber-optic cable through which the sample in the spectrophotometer was irradiated from the top.

Synthetic Procedures

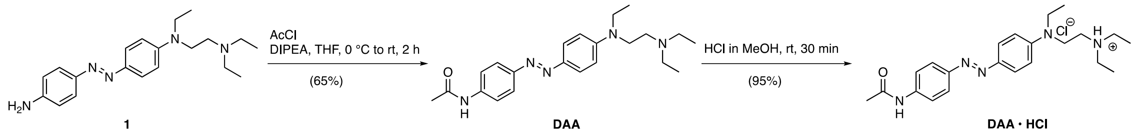

Supplementary Scheme 1. Synthesis of DAA • HCl, starting from the known precursor **1.**[1]

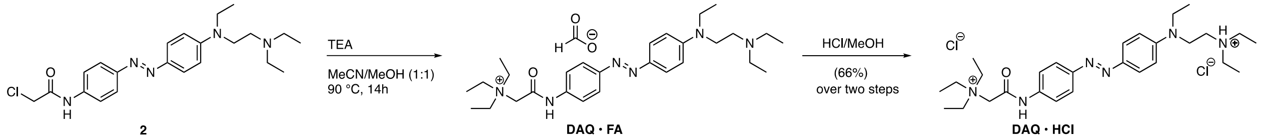

Supplementary Scheme 2. Synthesis of DAQ • HCl, starting from the known precursor **2.**[1]

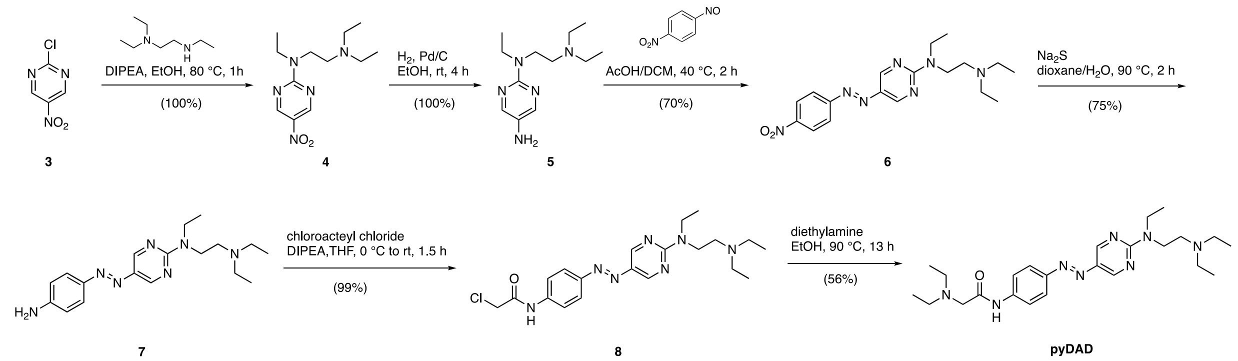

Supplementary Scheme 3. Synthesis of **pyDAD**.

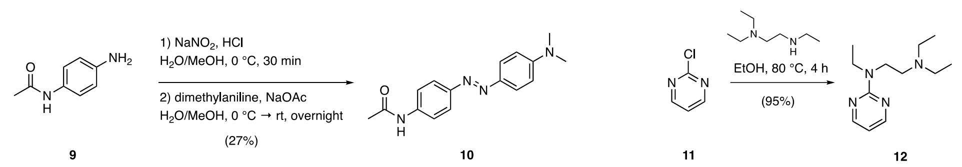

Supplementary Scheme 4. Synthesis of **10** and **12**.

(*E*)-*N*-(4-((4-((2-(diethylamino)ethyl)(ethyl)amino)phenyl)diazenyl)phenyl)acetamide (DAA)

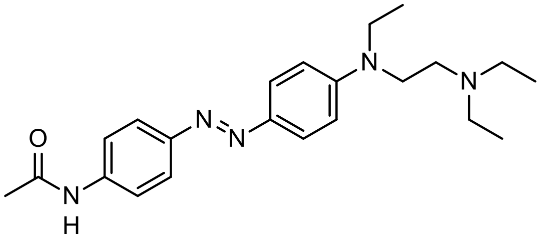

(*E*)-*N*1-(4-((4-aminophenyl)diazenyl)phenyl)-N^1,N^2,N^2-triethylethane-1,2-diamine (**1**) was synthesized according to literature.[1] **1** (20.0 mg, 58.9 µM, 1.0 eq) was dissolved in THF (1 mL) and DIPEA (12.2 µL, 70.7 µmol, 1.2 eq) and acetyl chloride (6.31 µL, 88.4 µmol, 1.5 eq) were added at 0°C. The reaction mixture was stirred for 2 h upon which it was allowed to warm up to room temperature. The reaction mixture was concentrated under reduced pressure and the crude mixture was purified by silica gel column chromatography (0.1% TEA + 0 → 5% MeOH/DCM). **DAA** (14.7 mg, 38.9 µmol, 65%) was gained as orange solid.

^{1}H NMR: (400 MHz, $CDCl_3$) δ = 7.87 – 7.75 (m, 4H), 7.62 (d, J = 8.8 Hz, 2H), 7.40 (s, 1H), 6.77 – 6.69 (m, 2H), 3.48 (m, 4H), 2.63 (m, 6H), 2.20 (s, 3H), 1.22 (t, J = 7.1 Hz, 3H), 1.07 (t, J = 7.1 Hz, 6H).
^{13}C NMR: (100 MHz, $CDCl_3$) δ = 168.4, 150.1, 149.8, 143.4, 139.0, 125.3, 123.2, 119.9, 111.1, 50.3, 49.5, 47.7, 45.8, 24.9, 12.6, 12.0.
HRMS (ESI): calcd for $C_{22}H_{32}N_5O^+$ $[M+H]^+$: 382.2601, found: 382.2607
R_f = 0.14 (5% MeOH/DCM)
λ_{max} = 448 nm

(*E*)-2-((4-((4-acetamidophenyl)diazenyl)phenyl)(ethyl)amino)-*N*,*N*-diethylethan-1-aminium chloride (DAA • HCl)

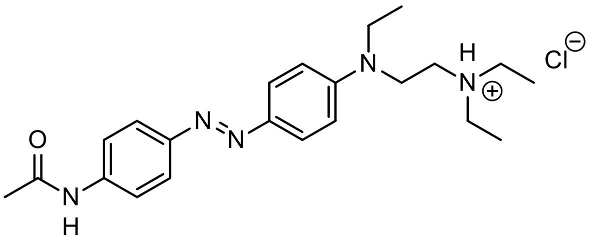

HCl in MeOH (1.25 M, 180 µL, 0.224 mmol, 1.5 eq) was added dropwise to a solution of **DAA** (57.1 mg, 0.150 mmol, 1.0 eq) in MeOH (1 mL) at rt, upon which the reaction mixture turned purple and was stirred for 5 min. The solvent was removed under reduced pressure and the product was dried under high vacuum to yield **DAA • HCl** (62.1 mg, 0.149 mmol, 99%) as dark orange solid.

^{1}H NMR: (400 MHz, DMSO-d_6) δ = 10.79 (s, 1H), 10.29 (s, 1H), 7.79 – 7.71 (m, 6H), 6.99 – 6.92 (m, 2H), 3.87 (t, J = 7.9 Hz, 2H), 3.50 (q, J = 6.9 Hz, 2H), 3.25 – 3.08 (m, 6H), 2.08 (s, 3H), 1.25 (t, J = 7.2 Hz, 6H), 1.15 (t, J = 6.9 Hz, 3H).
^{13}C NMR: (100 MHz, DMSO) δ = 168.7, 149.3, 147.8, 142.9, 141.0, 124.7, 122.7, 119.15, 111.8, 47.6, 46.2, 44.6, 44.2, 24.2, 12.2, 8.4.
HRMS (ESI): calcd for $C_{22}H_{32}N_5O^+$ [M+H]$^+$: 382.2601, found: 382.2598
R_t = 4.848 min (10 → 100% MeCN/H_2O + 0.1% FA, 10 min)
λ_{max} = 448 nm

(*E*)-2-((4-((4-((2-(diethylamino)ethyl)(ethyl)amino)phenyl)diazenyl)phenyl)amino)-*N*,*N*,*N*-triethyl-2-oxoethan-1-aminium (DAQ • HCl)

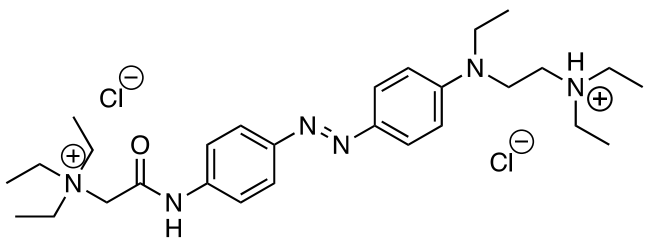

(*E*)-2-chloro-N-(4-((4-((2-(diethylamino)ethyl)(ethyl)amino)phenyl)diazenyl)phenyl)acet-amide (**2**) was prepared according to the literature.[1] **2** (41.0 mg, 98.6 µmol, 1.0 eq) was dissolved in MeCN/MeOH (1.5 mL, 1:1), triethylamine (690 µL) was added and the reaction mixture was heated to 90°C in a sealed flask overnight. All solvents were removed under reduced pressure and the crude material was purified by reverse-phase flash column chromatography (10% → 100% MeCN/H_2O + 0.1% FA) to yield **DAQ** as the formate salt. Addition of HCl in MeOH (1.25 M, 790 µL, 10.0 eq) followed by removal of the solvent in vacuo yielded **DAQ • HCl** (35.8 mg, 0.0647 mmol, 66%) as deep purple solid.

^{1}H NMR: (400 MHz, DMSO-d_6) δ = 11.98 (s, 1H), 11.30 (s, 1H), 7.89 (d, J = 8.9 Hz, 2H), 7.82 – 7.76 (m, 4H), 7.00 (d, J = 9.2 Hz, 2H), 4.46 (s, 2H), 3.96 – 3.89 (m, 2H), 3.56 – 3.49 (m, 8H), 3.24 -3 12 (m, 6H), 1.32 – 1.24 (m, 15H), 1.15 (t, J = 6.9 Hz, 3H).
^{13}C NMR: (100 MHz, DMSO-d_6) δ = 162.2, 149.6, 148.7, 142.9, 139.5, 124.9, 122.7, 120.0, 111.8, 56.5, 54.2, 47.5, 46.0, 44.6, 44.2, 12.2, 8.3, 7.6.
HRMS (ESI): calcd for $C_{28}H_{45}N_6O^+$ [M]+: 481.3649, found: 481.3659
R_t = 4.972 min (10 → 100% MeCN/H_2O + 0.1% FA, 10 min)
λ_{max} = 448 nm

N^1,N^1,N^2-triethyl-N^2-(5-nitropyrimidin-2-yl)ethane-1,2-diamine (4)

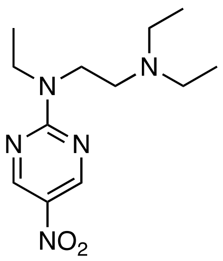

2-Chloro-5-nitropyrimidine (**3**) (100 mg, 0.627 mmol, 1.0 eq.) was dissolved in EtOH (2 mL). N^1,N^2,N^2-triethylethane-1,2-diamine (124 µL, 0.690 mmol, 1.1 eq.) and DIPEA (119 µL, 0.690 mmol, 1.1 eq.) were added and heated to 80°C for 2h in a sealed flask. The reaction mixture was diluted with $NaHCO_3$ (20 mL) and extracted with EtOAc (3 x 20 mL). The combined organic layers were washed with NaOH (1M, 2 x 20 mL) and sat. NaCl (20 mL) and dried over Na_2SO_4. The solvent was removed *in vacuo*. The product (**4**) (168 mg, 0.627 mmol, quant.) was obtained as a pale, yellow oil and used without further purification.

^{1}H NMR: (400 MHz, $CDCl_3$) δ = 9.05 (s, 2H), 3.79 – 3.69 (m, 4H), 2.69 – 2.63 (m, 2H), 2.58 (q, *J* = 7.1 Hz, 4H), 1.22 (t, *J* = 7.1 Hz, 3H), 1.03 (t, *J* = 7.1 Hz, 6H).
^{13}C NMR: (100 MHz, $CDCl_3$) δ = 161.6, 154.9, 133.4, 50.4, 47.7, 47.1, 44.4, 12.8, 12.2.
HRMS (ESI): calcd for $C_{12}H_{22}N_5O_2^+$ [M+H]$^+$: 268.1768, found: 268.1768.
R_f = 0.6 (10% MeOH/DCM, TLC deactivated with ammonia)

***N*²-(2-(diethylamino)ethyl)-*N*²-ethylpyrimidine-2,5-diamine (5)**

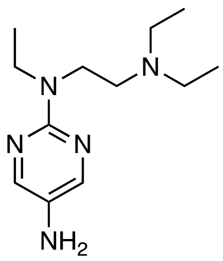

Pd/C (33.4 mg, 0.313 mmol, 0.5 eq.) was wetted with EtOH and **4** (168 mg, 0.627 mmol, 1.0 eq.) dissolved EtOH (5 mL) was added. The reaction vessel was purged with H_2 and a H_2 balloon was placed on top. The reaction was stirred at room temperature for 4 h. The crude material was filtered over celite and the solvent was removed under reduced pressure. The product (**5**) (148 mg, 0.624 mmol, quant) was obtained as a brown oil and used without further purification.

^{1}H NMR: (400 MHz, $CDCl_3$) δ = 7.96 (s, 2H), 3.66 – 3.53 (m, 4H), 3.02 (s, 2H), 2.67 – 2.60 (m, 6H), 1.15 (t, J = 7.0 Hz, 3H), 1.08 (t, J = 7.1 Hz, 6H).
^{13}C NMR: (100 MHz, $CDCl_3$) δ = 157.2, 147.0, 129.8, 50.5, 47.6, 45.8, 43.1, 13.3, 11.8.
HRMS (ESI): calcd for $C_{12}H_{24}N_5^+$ $[M+H]^+$: 238.2026, found: 238.2031.
R_f = 0.2 (10% MeOH/DCM, TLC deactivated with ammonia)

(*E*)-*N*[1],*N*[1],*N*[2]-triethyl-*N*[2]-(5-((4-nitrophenyl)diazenyl)pyrimidin-2-yl)ethane-1,2-diamine (6)

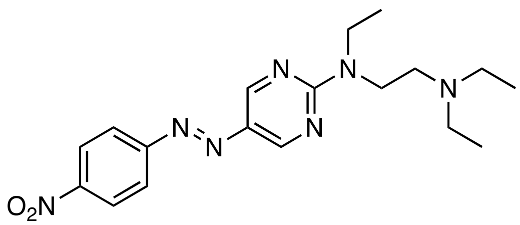

5 (150 mg, 0.632 mmol, 1.0 eq) and 1-nitro-4-nitrosobenzene (111 mg, 0.632 mmol, 1.15 eq.) were dissolve in DCM/AcOH (1:1, 6 mL) and stirred at 40°C for 4 h. The reaction mixture was neutralized with NaOH (1M) and extracted with EtOAc. The combined organic layers were washed with sat. NaCl, dried over Na_2SO_4 and the solvent was removed *in vacuo*. The crude material was purified by silica gel column chromatography (NH_3 deactivated silica gel, 0% → 1% → 10% MeOH/DCM) to yield the product (**6**) (164 mg, 0.440 mmol, 70%) as red solid.

^{1}H NMR: (400 MHz, $CDCl_3$) δ = 8.91 (s, 2H), 8.35 (d, J = 9.0 Hz, 2H), 7.94 (d, J = 9.0 Hz, 2H), 3.80 – 3.75 (m, 4H), 2.77 – 2.69 (m, 2H), 2.63 (q, J = 7.1 Hz, 4H), 1.26 (t, J = 7.1 Hz, 4H), 1.08 (t, J = 7.1 Hz, 6H).
^{13}C NMR: (100 MHz, $CDCl_3$) δ = 161.8, 156.3, 154.5, 153.9, 148.1, 137.9, 124.9, 123.1, 50.5, 47.7, 46.7, 44.1, 13.1, 12.1.

Note: carbons at 154.5 and 153.9 couple to the proton signal at 8.91.

HRMS (ESI): calcd for $C_{18}H_{26}N_7O_2^+$ $[M+H]^+$: 372.2142, found: 372.2151.
R_f = 0.4 (10% MeOH/DCM).

(*E*)-*N*[1]-(5-((4-aminophenyl)diazenyl)pyrimidin-2-yl)-*N*[1],*N*[2],*N*[2]-triethylethane-1,2-diamine (7)

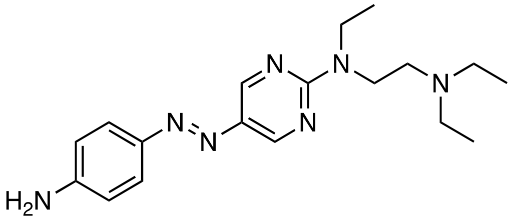

6 (160 mg, 0.431 mmol, 1.0 eq.) was dissolved in 1,4-dioxane (12 mL), Na_2S (84.1 mg, 1.08 mmol, 2.5 eq.) in water (1 mL) was added and the reaction was heated to 95 °C for 3 h. The reaction mixture was diluted with EtOAc (30 mL) and washed with water (2 x 10 mL), 10% NaCl (2 x 10 mL), aq. NaOH (1M, 2 x 10 mL) and sat. NaCl (1 x 10 mL). The organic phase was dried over Na_2SO_4 and the solvent was removed *in vacuo*. The crude material was purified by silica gel column chromatography (NH_3 deactivated silica gel, 0% → 5% MeOH/DCM) to yield the product (**7**) (139 mg, 0.407 mmol, 95%) as orange solid.

^{1}H NMR: (400 MHz, $CDCl_3$) δ = 8.80 (s, 2H), 7.71 (d, J = 8.7 Hz, 2H), 6.72 (d, J = 8.8 Hz, 2H), 4.03 (s, 2H), 3.82 – 3.68 (m, 4H), 2.80 – 2.73 (m, 2H), 2.69 (q, J = 7.1 Hz, 4H), 1.23 (t, J = 7.0 Hz, 3H), 1.12 (t, J = 7.1 Hz, 6H).
^{13}C NMR: (100 MHz, $CDCl_3$) δ = 161.1, 152.8, 149.1, 145.8, 137.9, 124.6, 114.8, 50.3, 47.6, 46.0, 43.8, 13.2, 11.7.
HRMS (ESI): calcd for $C_{18}H_{28}N_7^+$ $[M+H]^+$: 342.2401, found: 342.2400.
R_f = 0.2 (10% MeOH/DCM)

(*E*)-2-chloro-*N*-(4-((2-((2-(diethylamino)ethyl)(ethyl)amino)pyrimidin-5-yl)diazenyl)phenyl)acetamide (8)

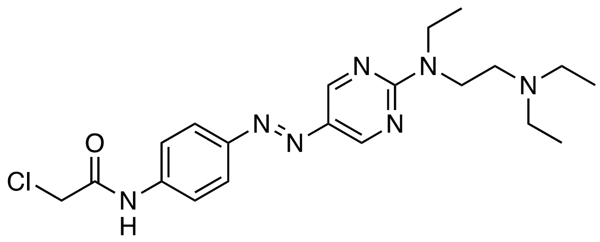

7 (136 mg, 0.398 mmol, 1.0 eq) was dissolved in THF (20 mL) and cooled to 0 °C. DIPEA (103 µL, 0.597 mmol, 1.2 eq.) was added and chloroacetyl chloride (38.0 µL, 0.478 mmol, 1.5 eq.) was added dropwise. The reaction was stirred at 0 °C for 30 min, upon which it was allowed to warm up to room temperature and stirred for 1.5 h. The reaction was quenched by addition of $NaHCO_3$ (10 mL) and extracted with EtOAc (3 x 10 mL). The combined organic layers were washed with NaOH (1M, 2 x 10 mL), $NaHCO_3$ (1 x 10 mL) and sat. NaCl (1 x 10 mL) and dried over Na_2SO_4. The solvent was removed *in vacuo* and the crude material was purified by column chromatography (NH_3 deactivated silica, 1% → 10% MeOH/DCM) to yield the product (**8**) (84.2 mg, 0.201 mmol, 51%) as orange solid.

^{1}H NMR: (400 MHz, $CDCl_3$) δ = 8.86 (s, 2H), 8.40 (s, 1H), 7.86 (d, J = 8.8 Hz, 2H), 7.70 (d, J = 8.9 Hz, 2H), 4.23 (s, 2H), 3.75 (q, J = 8.6, 7.1 Hz, 4H), 2.77 – 2.68 (m, 2H), 2.64 (q, J = 7.1 Hz, 4H), 1.25 (t, J = 7.1 Hz, 3H), 1.09 (t, J = 7.1 Hz, 6H).
^{13}C NMR: (100 MHz, $CDCl_3$) δ = 163.9, 161.5, 153.3, 150.0, 138.5, 137.8, 123.6, 120.3, 50.5, 47.7, 46.5, 43.9, 43.1, 13.1, 12.0.
Note: the signal at 153.3 was extracted from the HSQC spectrum.
HRMS (ESI): calcd for $C_{20}H_{29}ClN_7O^+$ $[M+H]^+$: 418.2117, found: 418.2120.
R_f = 0.2 (10% MeOH/EtOAc)

(*E*)-2-(diethylamino)-*N*-(4-((2-((2-(diethylamino)ethyl)(ethyl)amino)pyrimidin-5-yl)diazenyl)phenyl)acetamide (pyDAD)

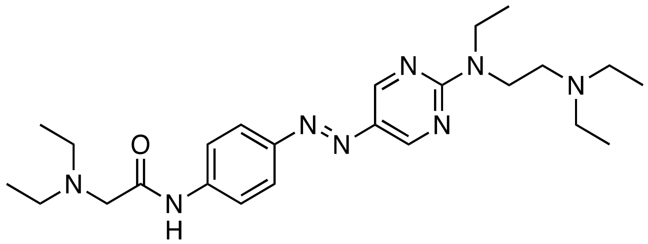

8 (33.8 mg, 80.8 µmol, 1.0 eq) was dissolved in EtOH (1 mL), diethylamine (83.2 µL, 0.808 mmol, 10 eq.) was added and the reaction was heated to 100 °C in a sealed flask overnight. The solvent was removed under reduced pressure and the crude material was purified by column chromatography (NH_3 deactivated silica gel, 25% acteone/$CHCl_3$) to yield **pyDAD** (20.4 mg, 44.9 µmol, 56%) as orange solid.

^{1}H NMR: (400 MHz, $CDCl_3$) δ = 9.60 (s, 1H), 8.85 (s, 2H), 7.84 (d, J = 8.8 Hz, 2H), 7.71 (d, J = 8.9 Hz, 2H), 3.77 – 3.60 (m, 4H), 3.18 (s, 2H), 2.71 – 2.53 (m, 10H), 1.24 (t, J = 7.1 Hz, 3H), 1.17 – 1.00 (m, 12H).
Note: Due to overlap of the signals, *trans* and *cis* (ratio 10:1) have been integrated here.
^{13}C NMR: (100 MHz, $CDCl_3$) δ = 170.5, 161.4, 153.2, 149.3, 139.7, 137.8, 123.6, 119.5, 58.3, 50.6, 49.0, 47.8, 46.6, 43.9, 13.2, 12.6, 12.2.
HRMS (ESI): calcd for $C_{24}H_{39}N_8O^+$ $[M+H]^+$: 455.3241, found: 455.3248.
R_f = 0.7 (20% MeOH/DCM)
λ_{max} = 382 nnm

(*E*)-*N*-(4-((4-(dimethylamino)phenyl)diazenyl)phenyl)acetamide

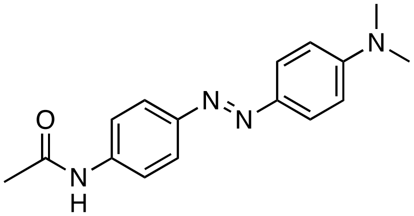

p-Aminoacetaniline (**9**) (200 mg, 1.33 mmol, 1.0 eq.) was dissolved in water/MeOH (1:1, 50 mL), aq. HCl (1M, 4.00 mL, 3.99 mmol, 3.0 eq) was added and the solution was cooled to 0 °C. $NaNO_2$ (110 mg, 1.46 mmol, 1.1 eq.) was added and the reaction was stirred at 0 °C for 30 min. This solution was then added dropwise to a solution of dimethylaniline and NaOAc (655 mg, 7.99 mmol, 6.0 eq.) in water/MeOH (1:1, 50 mL). The solution was stirred at 0 °C and allowed to warm up to room temperature overnight. The organic solvent was removed under reduced pressure and the aqueous phase was extracted with EtOAc (4 x 50 mL). The combined organic layers were washed with $NaHCO_3$ (3 x 50 mL) and sat. NaCl (1 x 50 mL). The organic layer was dried over Na_2SO_4 and the solvent was removed *in vacuo*. The crude material was purified by silica gel column chromatography (60% EtOAc/hexanes) to yield the product **10** (99.8 mg, 0.353 mmol, 27%) as orange solid.

^{1}H NMR: (400 MHz, $CDCl_3$) δ = 7.84 (t, 4H), 7.62 (d, 2H), 7.33 (s, 1H), 6.75 (d, 2H), 3.08 (s, 6H), 2.21 (s, 3H).
^{13}C NMR: (100 MHz, $CDCl_3$) δ = 168.3, 152.4, 149.8, 143.7, 139.1, 124.9, 123.3, 119.9, 111.7, 40.5, 24.9.
HRMS (ESI): calcd for $C_{24}H_{39}N_8O^+$ $[M+H]^+$: 455.3241, found: 455.3248.
R_f = 0.7 (20% MeOH/DCM)

N^1,N^1,N^2-triethyl-N^2-(pyrimidin-2-yl)ethane-1,2-diamine

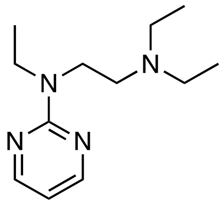

2-Chloropyrimidine (**11**) (100 mg, 0.873 mmol, 1.0 eq.) and N^1,N^2,N^2-triethylethane-1,2-diamine (783 µL, 4.37 mmol, 5.0 eq.) were dissolved in EtOH (2 mL) and heated to 80 °C for 4 h in a sealed flask. The solvent was removed under reduced pressure and the crude material was purified by silica gel column chromatography (0 → 5% MeOH/DCM) to yield the product (**12**) (185 mg, 0.832 mmol, 95%) as a pale white oil.

^{1}H NMR: (400 MHz, $CDCl_3$) δ = 8.27 (d, *J* = 4.7 Hz, 2H), 6.40 (t, *J* = 4.7 Hz, 1H), 3.68 – 3.58 (m, 4H), 2.68 – 2.56 (m, 6H), 1.18 (t, *J* = 7.1 Hz, 3H), 1.06 (t, *J* = 7.1 Hz, 6H).
^{13}C NMR: (100 MHz, $CDCl_3$) δ = 161.3, 157.8, 109.0, 50.5, 47.7, 45.9, 43.0, 13.1, 12.1.
HRMS (ESI): calcd for $C_{12}H_{23}N_4^+$ [M+H]$^+$: 223.1917, found: 223.1919
R_f = 0.3 (3% MeOH/DCM)

NMR spectra

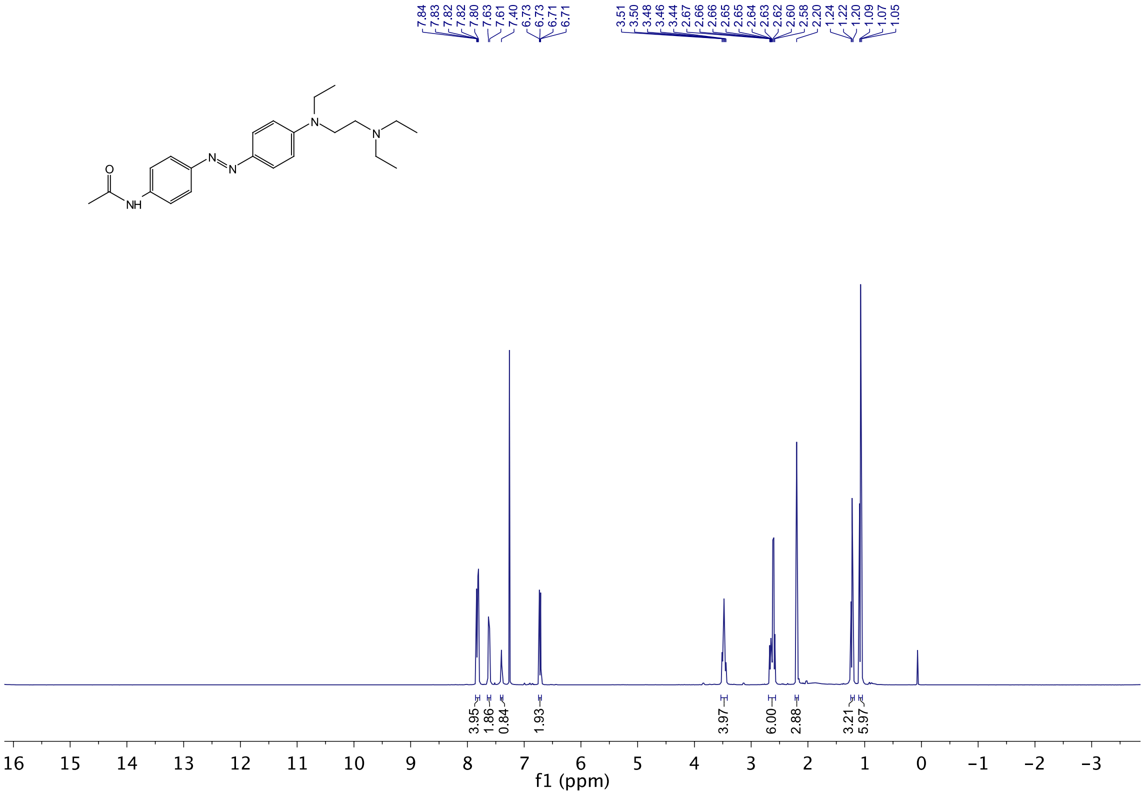
7.84
7.83
7.82
7.82
7.80
7.63
7.61
7.40
6.73
6.73
6.71
6.71
3.51
3.50
3.48
3.46
3.44
2.67
2.66
2.66
2.65
2.65
2.64
2.63
2.62
2.60
2.58
2.20
1.24
1.22
1.20
1.09
1.07
1.05
O
NH
N
N
N
N
3.95
1.86
0.84
1.93
3.97
6.00
2.88
3.21
5.97
16 15 14 13 12 11 10 9 8 7 6 5 4 3 2 1 0 −1 −2 −3
f1 (ppm)

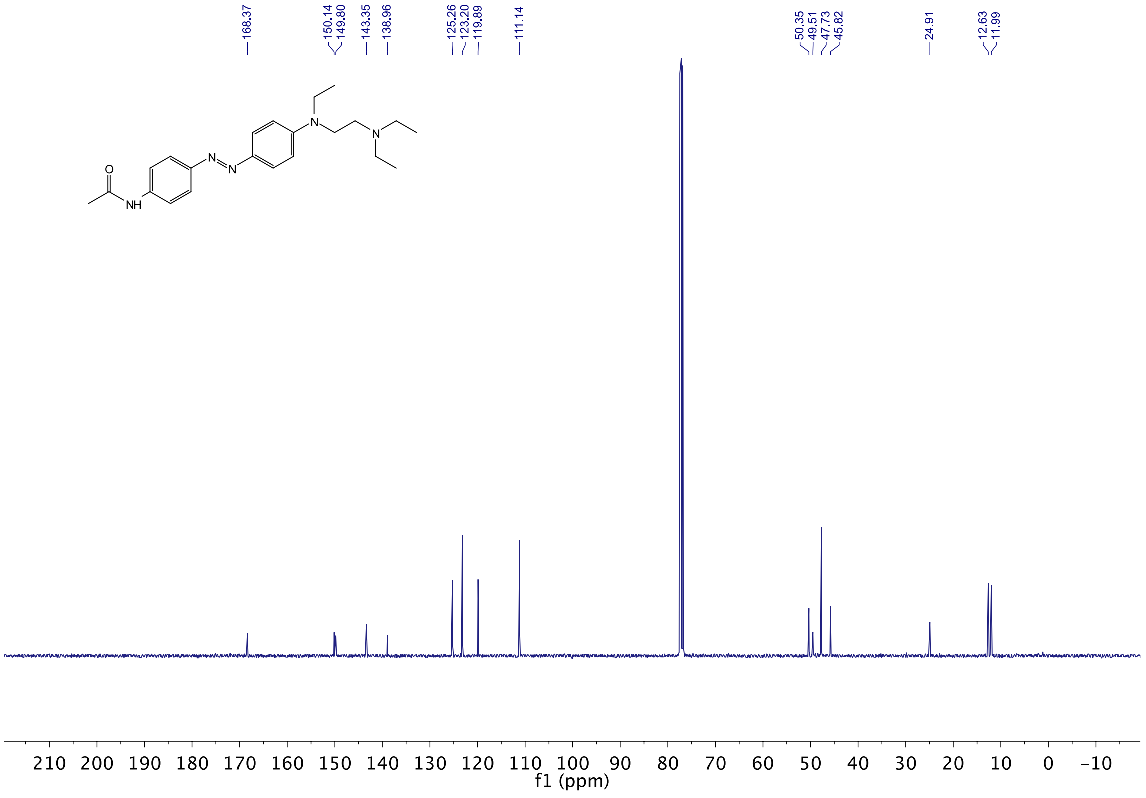
168.37
150.14
149.80
143.35
138.96
125.26
123.20
119.89
111.14
50.35
49.51
47.73
45.82
24.91
12.63
11.99
O
NH
N
N
N
N
210 200 190 180 170 160 150 140 130 120 110 100 90 80 70 60 50 40 30 20 10 0 −10
f1 (ppm)

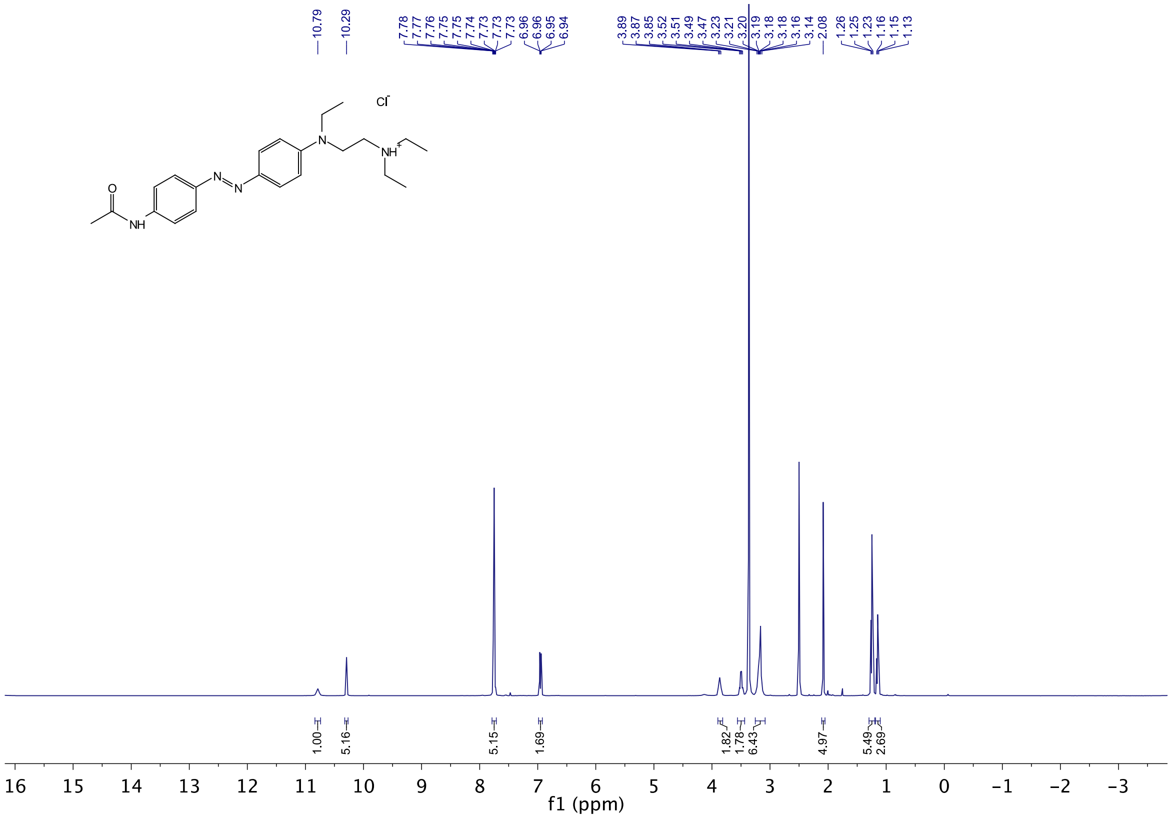

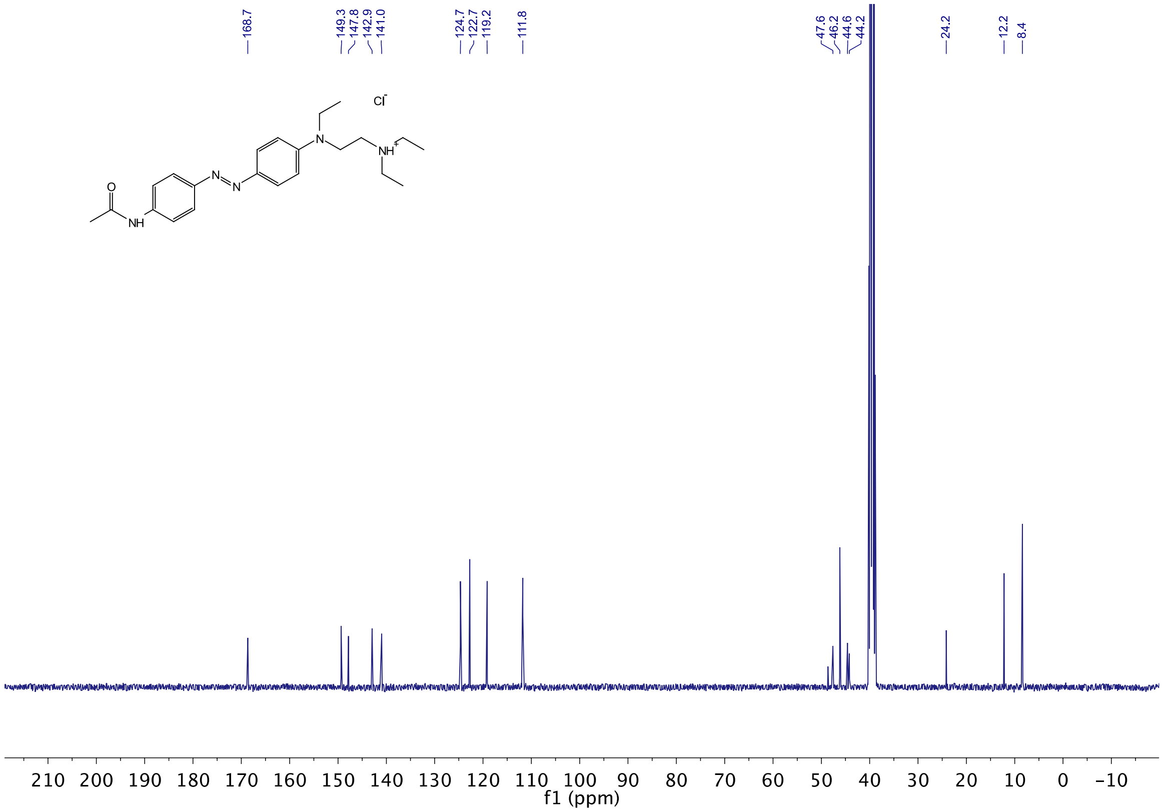

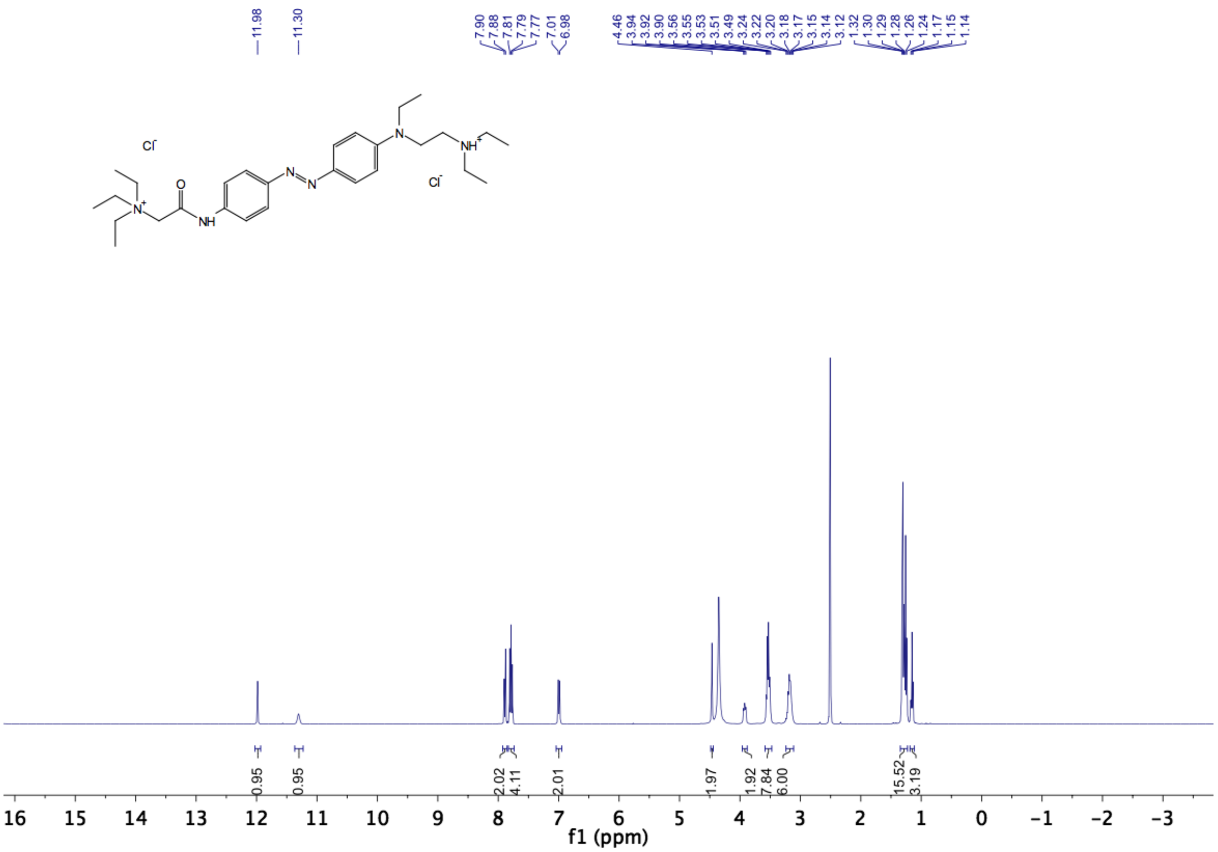
11.98
11.30
7.90
7.88
7.81
7.79
7.77
7.01
6.98
4.46
3.94
3.92
3.90
3.56
3.55
3.53
3.51
3.49
3.24
3.22
3.20
3.18
3.17
3.15
3.14
3.12
1.32
1.30
1.29
1.28
1.26
1.24
1.17
1.15
1.14
Cl-
Cl-
NH
NH+
O
N
N
N
N+
0.95
0.95
2.02
4.11
2.01
1.97
1.92
7.84
6.00
15.52
3.19
f1 (ppm)

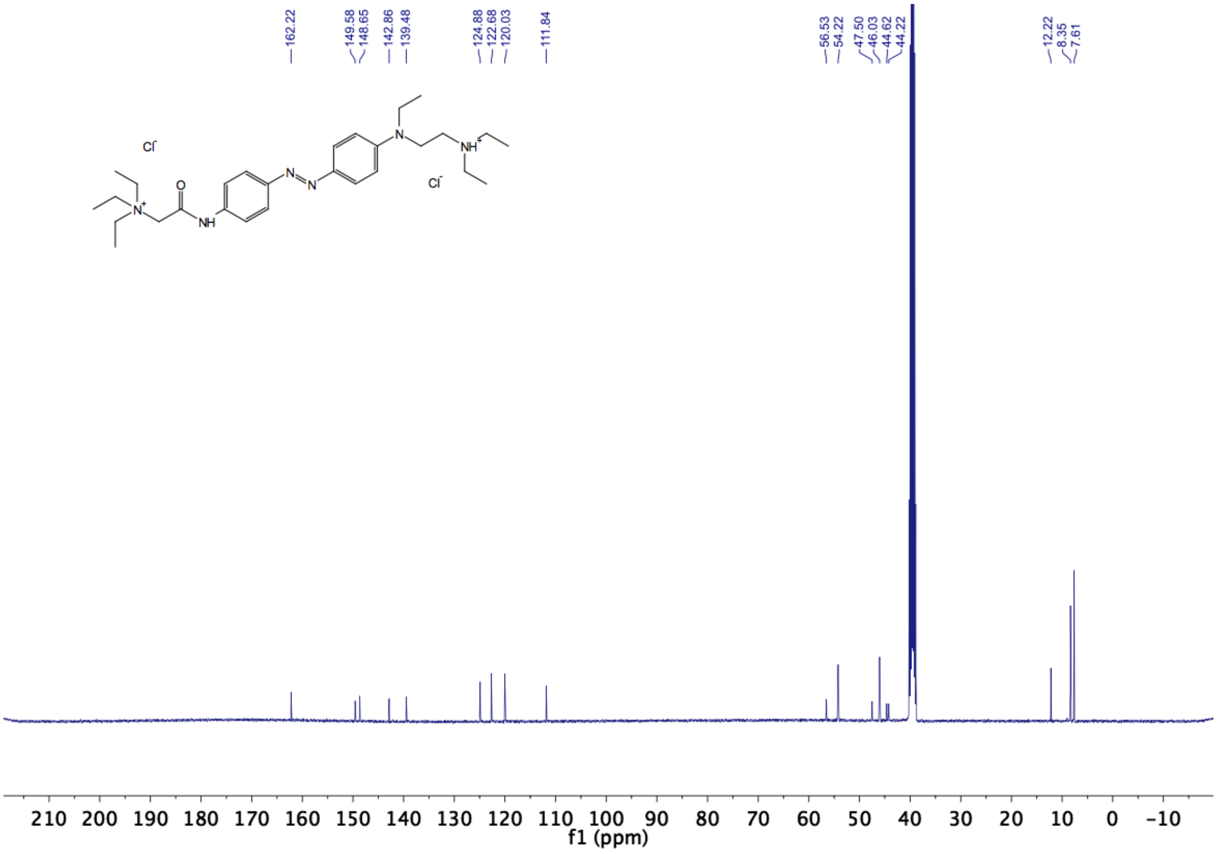
162.22
149.58
148.65
142.86
139.48
124.88
122.68
120.03
111.84
56.53
54.22
47.50
46.03
44.62
44.22
12.22
8.35
7.61
Cl-
Cl-
NH
NH+
O
N
N
N
N+
f1 (ppm)

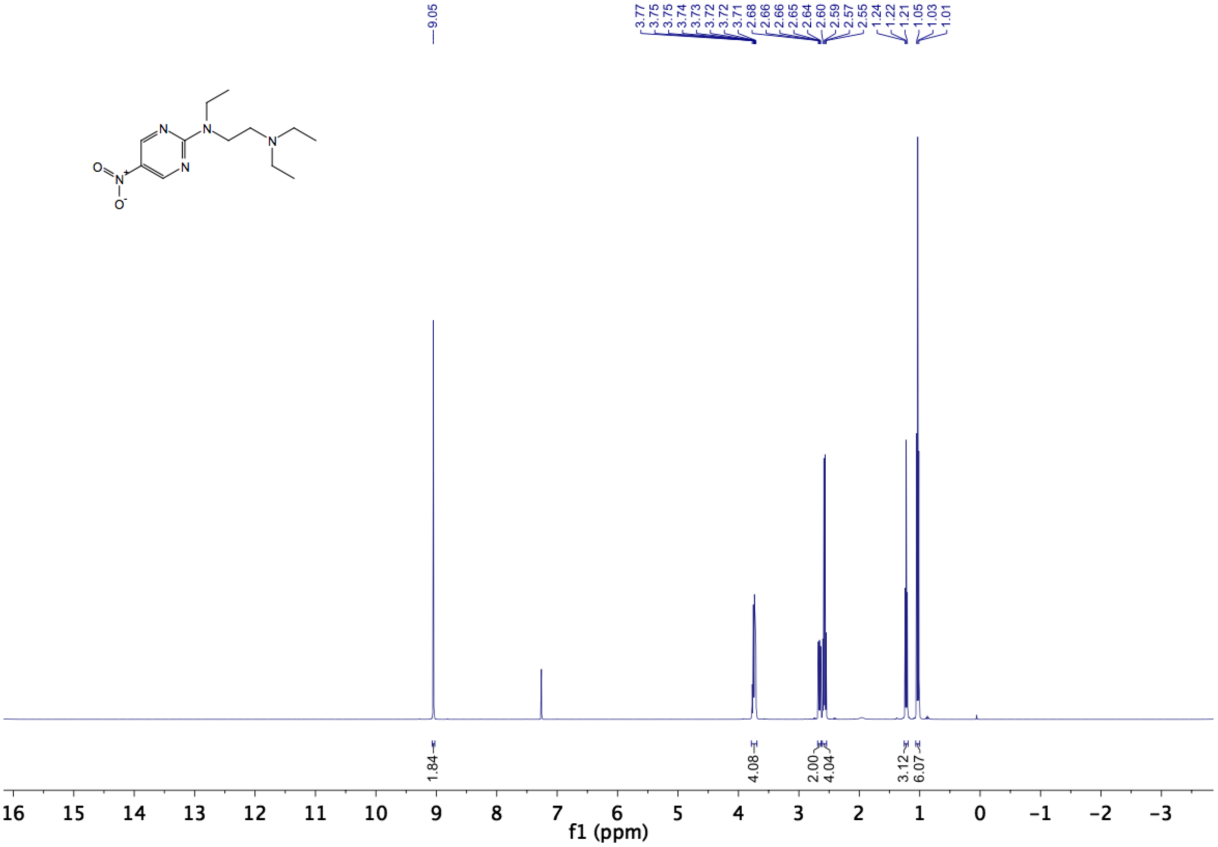
9.05
3.77
3.75
3.75
3.74
3.73
3.72
3.72
3.71
2.68
2.66
2.66
2.65
2.64
2.60
2.59
2.57
2.55
1.24
1.22
1.21
1.05
1.03
1.01
1.84
4.08
2.00
4.04
3.12
6.07
16 15 14 13 12 11 10 9 8 7 6 5 4 3 2 1 0 −1 −2 −3
f1 (ppm)

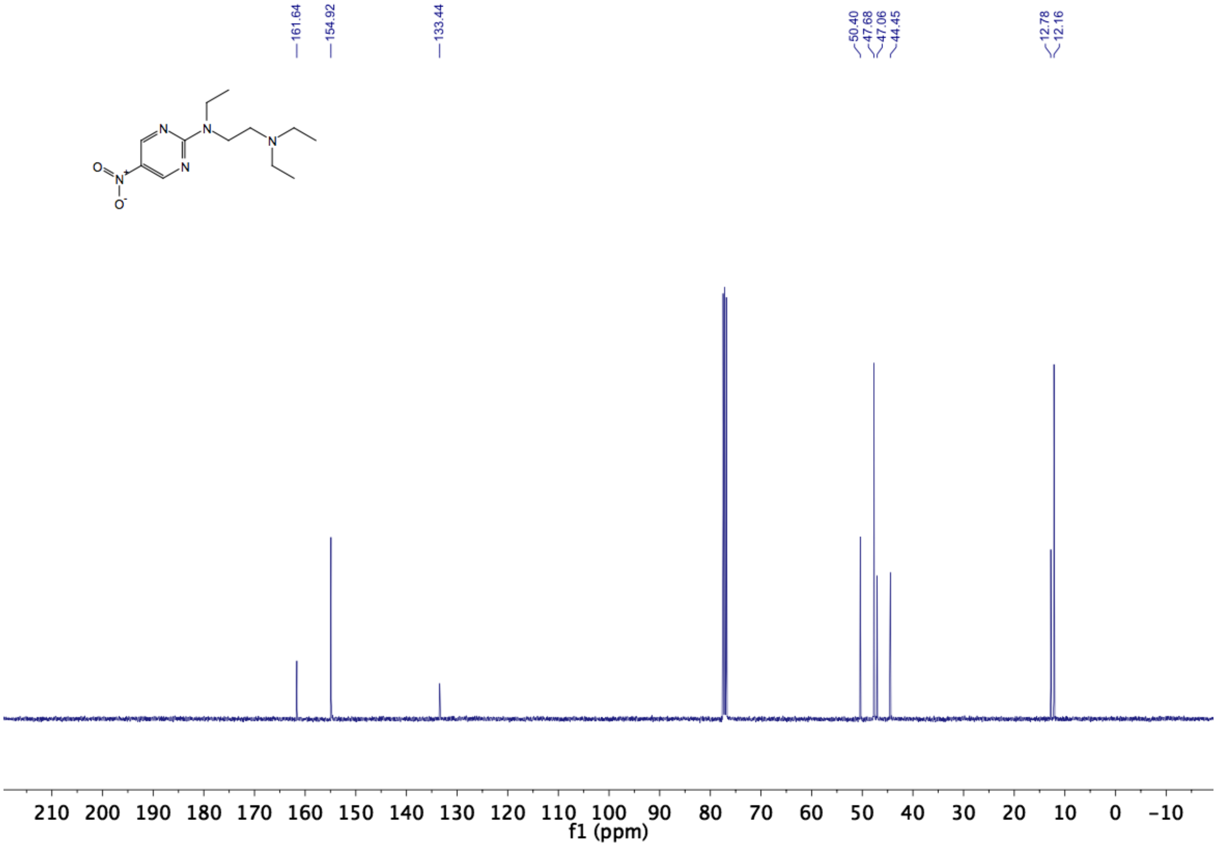
161.64
154.92
133.44
50.40
47.68
47.06
44.45
12.78
12.16
210 200 190 180 170 160 150 140 130 120 110 100 90 80 70 60 50 40 30 20 10 0 −10
f1 (ppm)

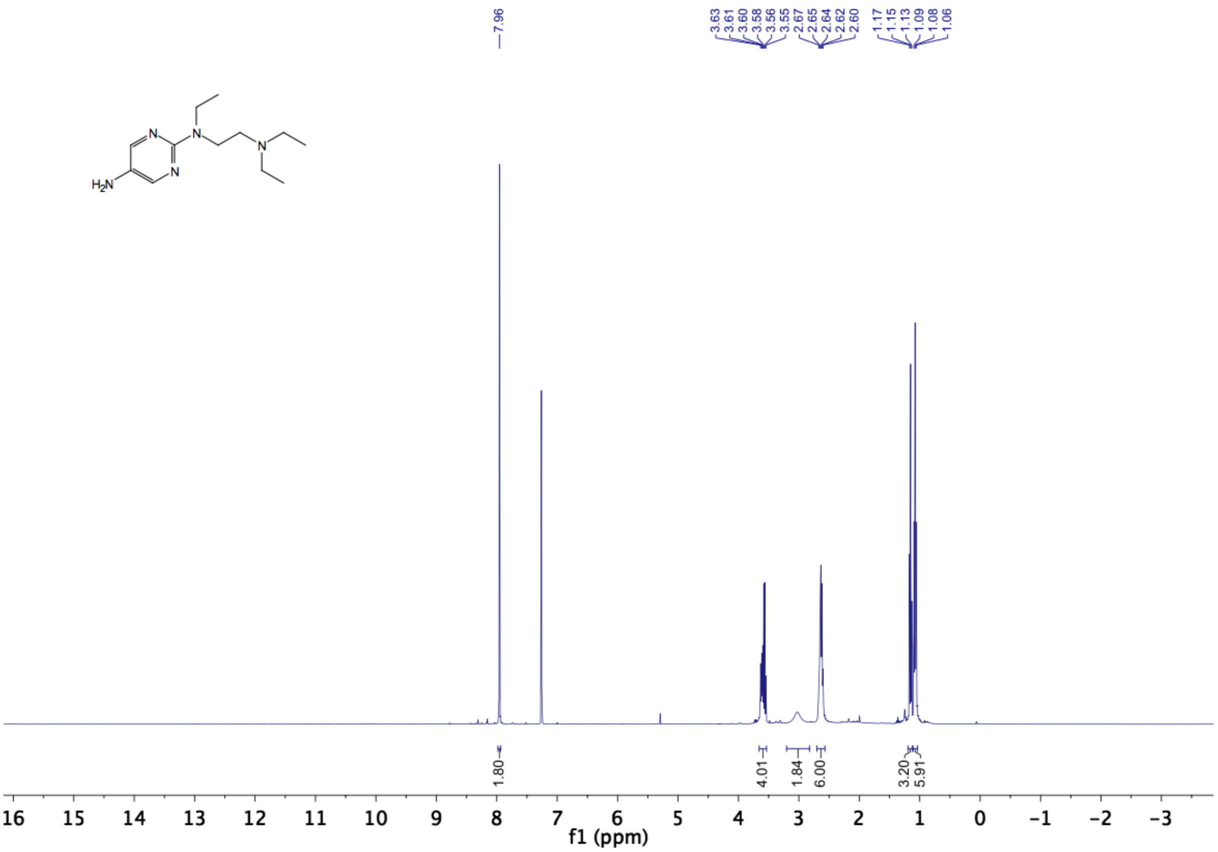
H2N
N
7.96
3.63
3.61
3.60
3.58
3.56
3.55
2.67
2.65
2.64
2.62
2.60
1.17
1.15
1.13
1.09
1.08
1.06
1.80
4.01
1.84
6.00
3.20
5.91
16 15 14 13 12 11 10 9 8 7 6 5 4 3 2 1 0 −1 −2 −3
f1 (ppm)

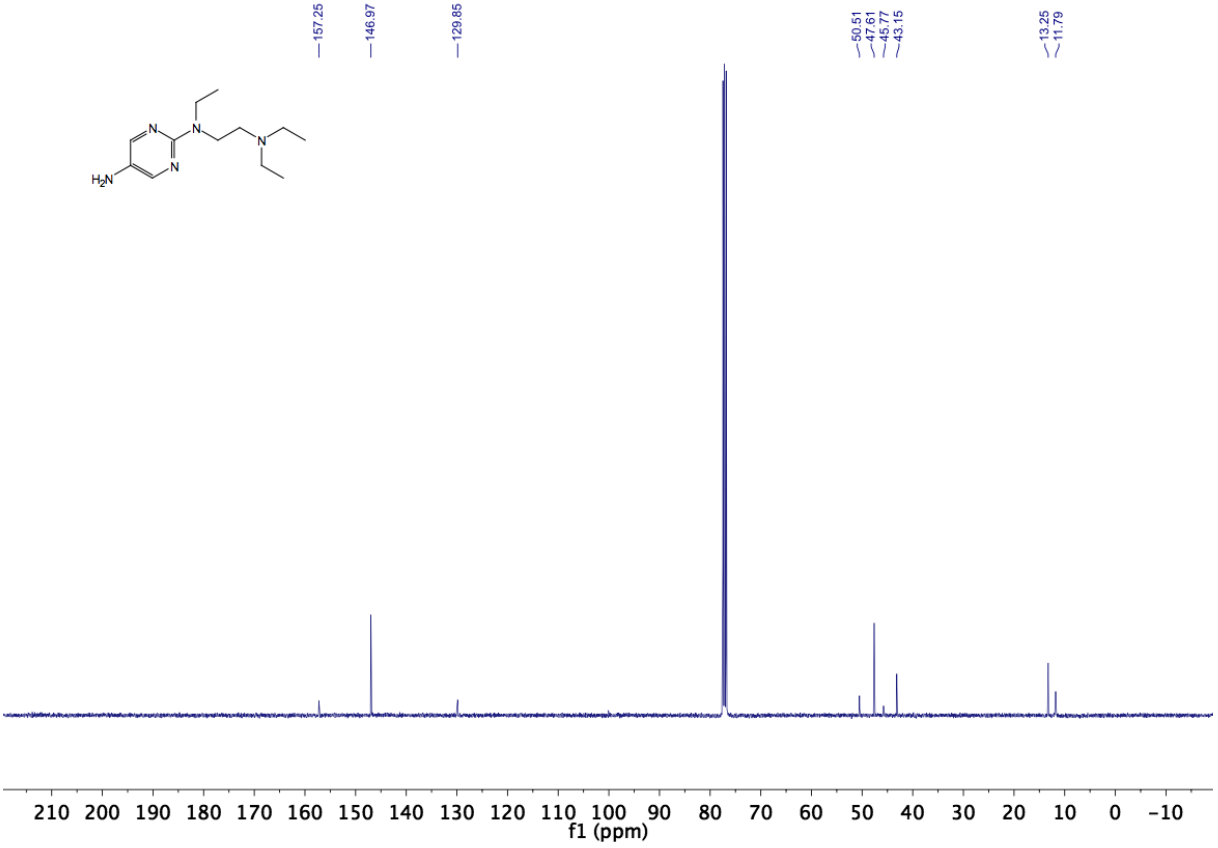
H2N
N
157.25
146.97
129.85
50.51
47.61
45.77
43.15
13.25
11.79
210 200 190 180 170 160 150 140 130 120 110 100 90 80 70 60 50 40 30 20 10 0 −10
f1 (ppm)

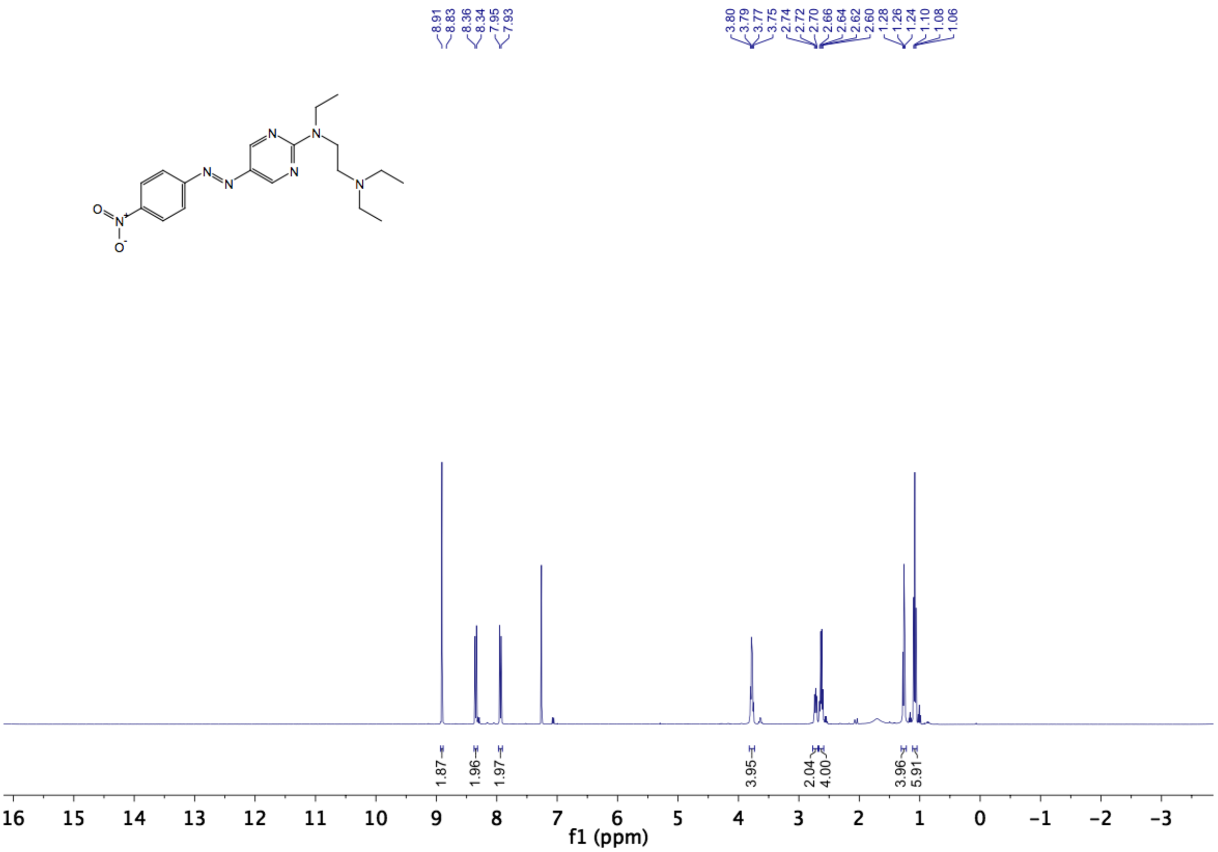

8.91
8.83
8.36
8.34
7.95
7.93
3.80
3.79
3.77
3.75
2.74
2.72
2.70
2.66
2.64
2.62
2.60
1.28
1.26
1.24
1.10
1.08
1.06
1.87
1.96
1.97
3.95
2.04
4.00
3.96
5.91
16 15 14 13 12 11 10 9 8 7 6 5 4 3 2 1 0 −1 −2 −3
f1 (ppm)

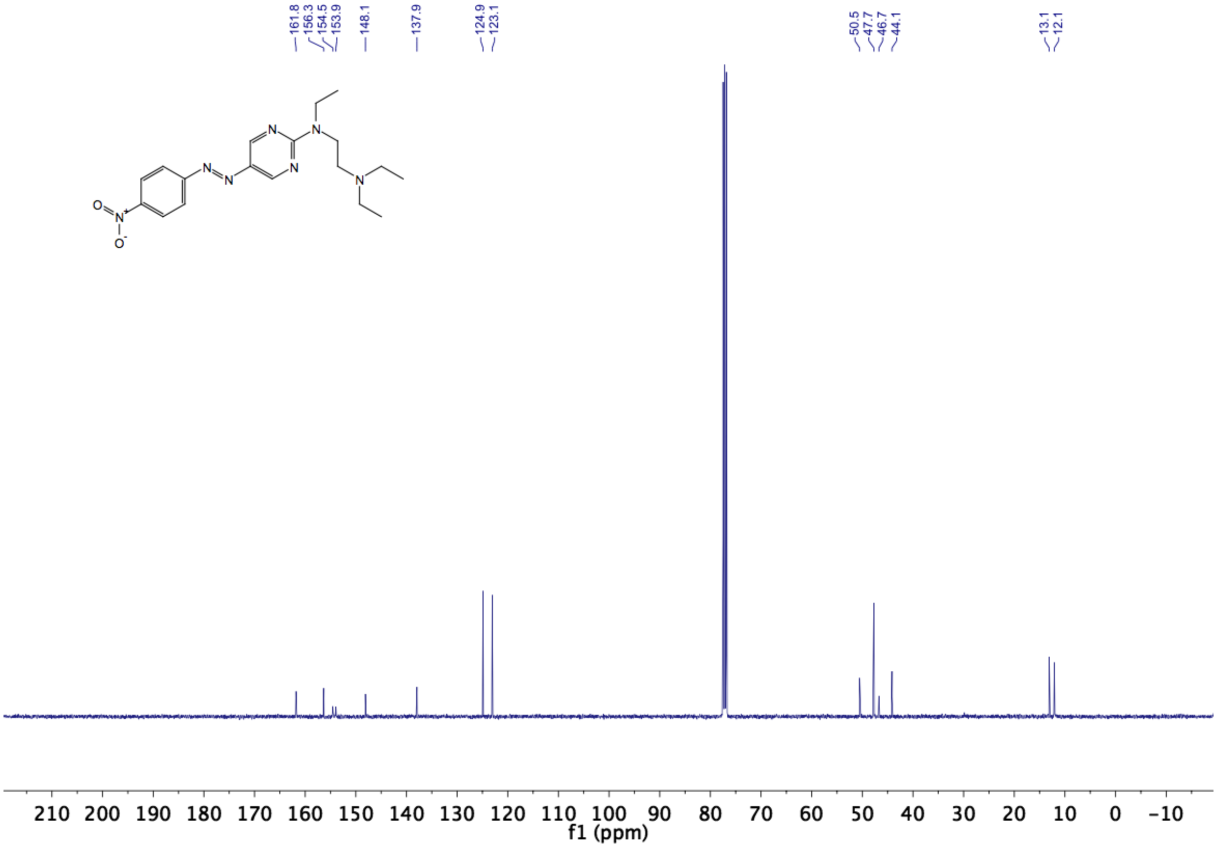

161.8
156.3
154.5
153.9
148.1
137.9
124.9
123.1
50.5
47.7
46.7
44.1
13.1
12.1
210 200 190 180 170 160 150 140 130 120 110 100 90 80 70 60 50 40 30 20 10 0 −10
f1 (ppm)

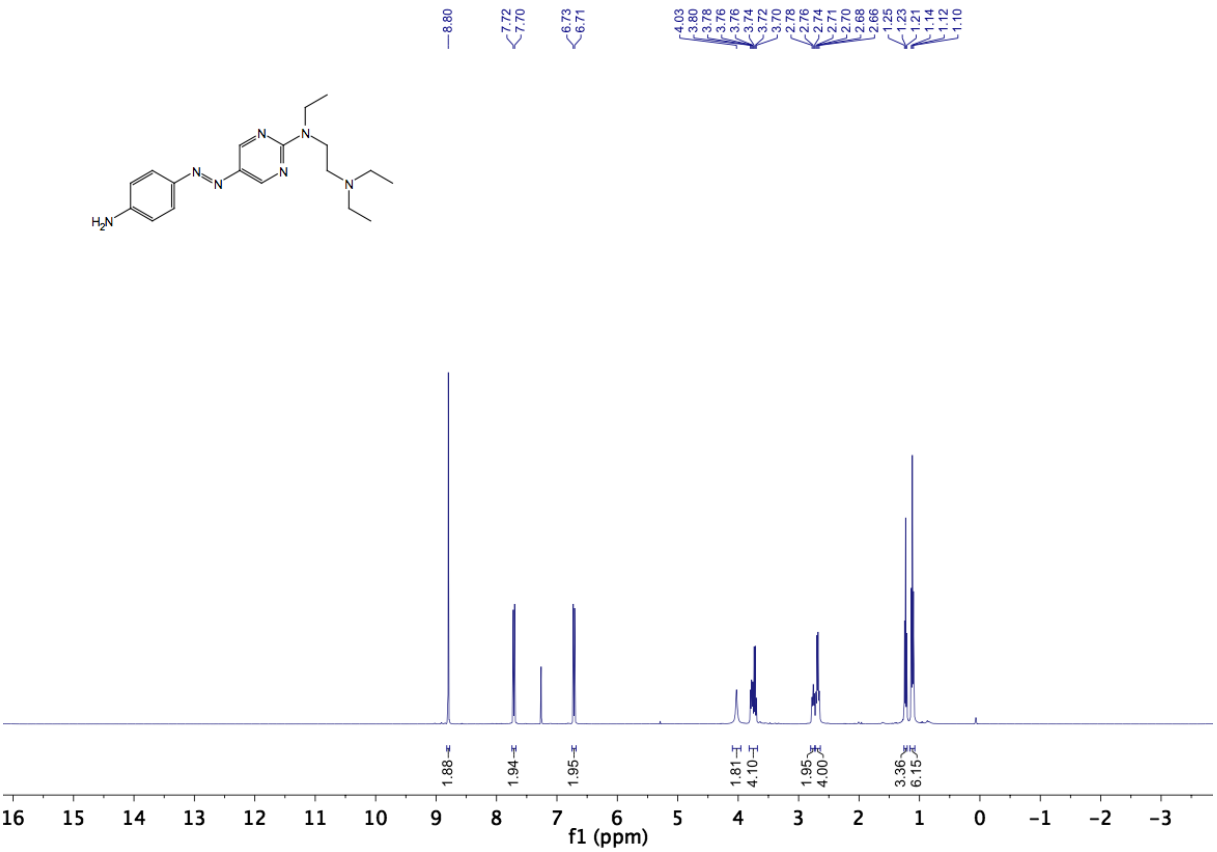

8.80
7.72
7.70
6.73
6.71
4.03
3.80
3.78
3.76
3.76
3.74
3.72
3.70
2.78
2.76
2.74
2.71
2.70
2.68
2.66
1.25
1.23
1.21
1.14
1.12
1.10
H2N
N
N
1.88
1.94
1.95
1.81
4.10
1.95
4.00
3.36
6.15
f1 (ppm)

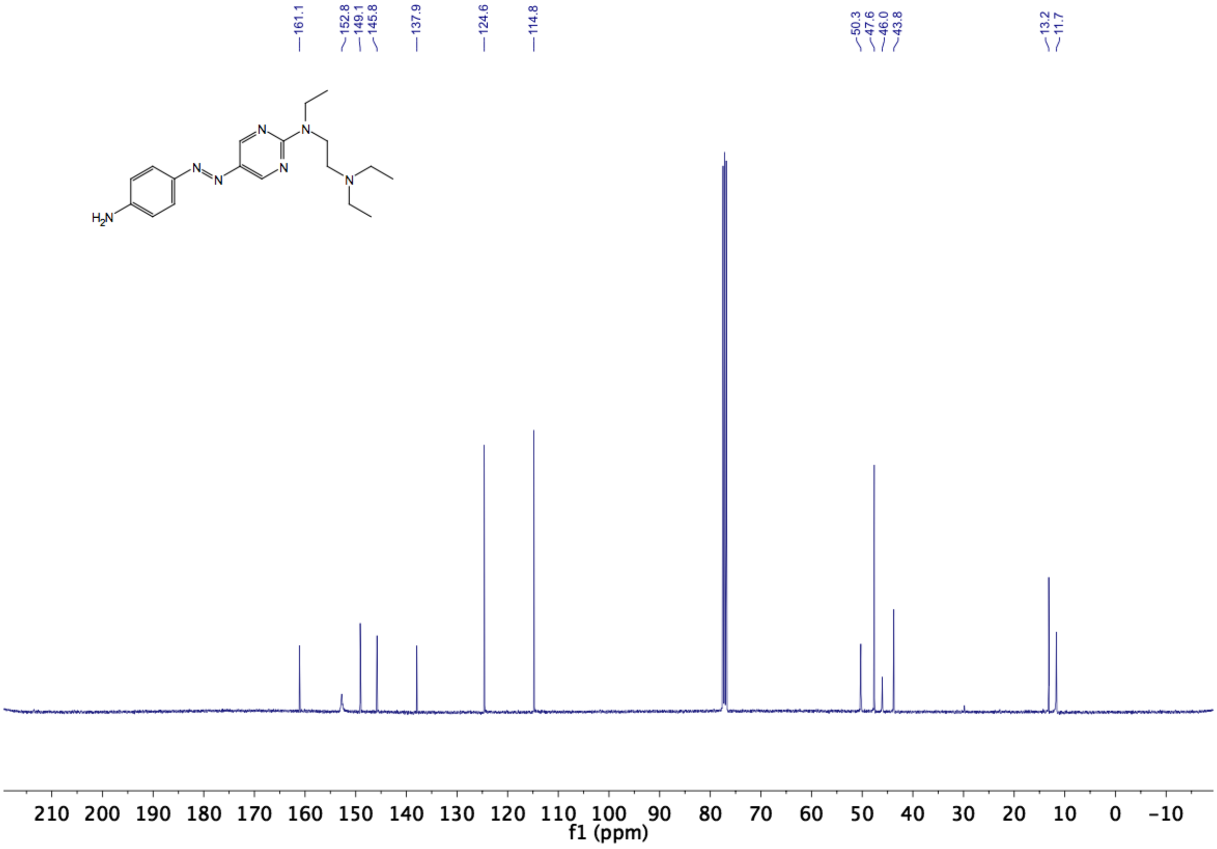

161.1
152.8
149.1
145.8
137.9
124.6
114.8
50.3
47.6
46.0
43.8
13.2
11.7
H2N
N
N
f1 (ppm)

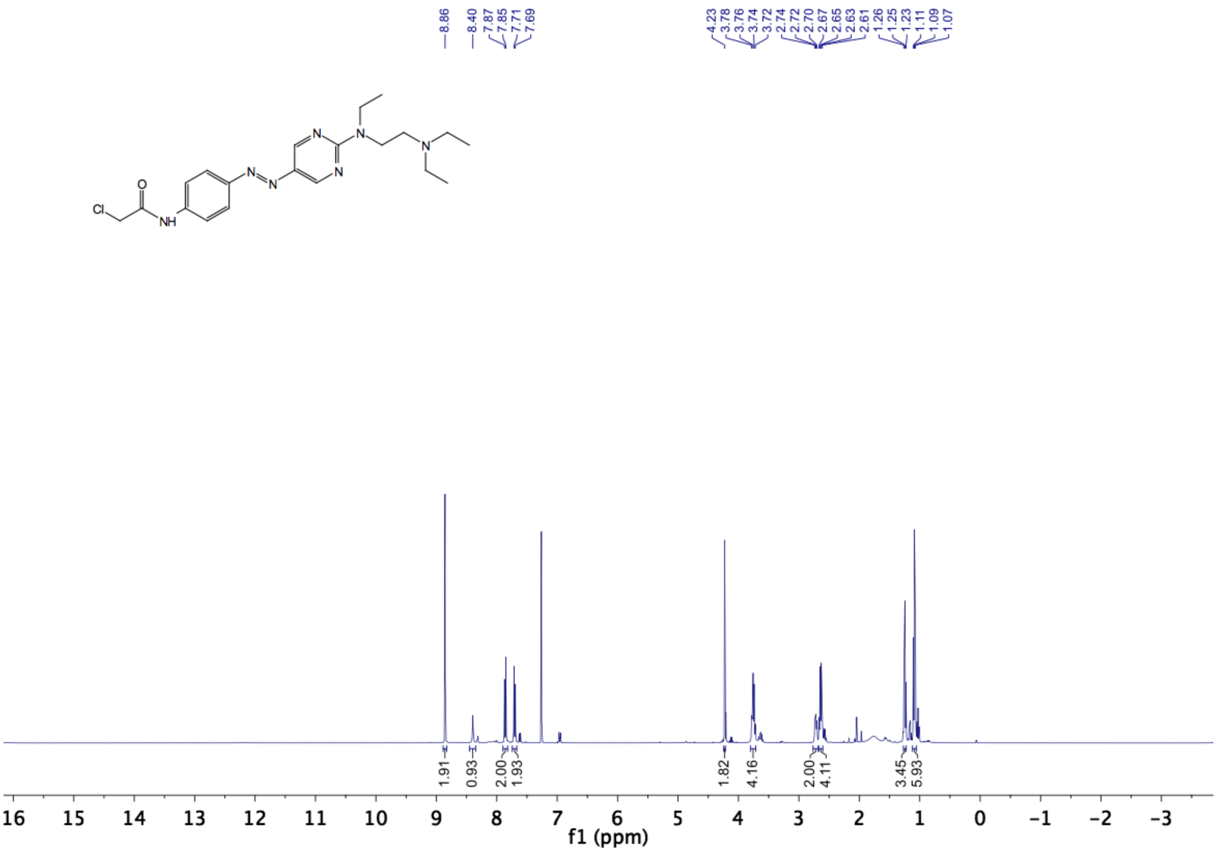
8.86
8.40
7.87
7.85
7.71
7.69
4.23
3.78
3.76
3.74
3.72
2.74
2.72
2.70
2.67
2.65
2.63
2.61
1.26
1.25
1.23
1.11
1.09
1.07
Cl
O
NH
N
N
N
N
N
N
1.91
0.93
2.00
1.93
1.82
4.16
2.00
4.11
3.45
5.93
f1 (ppm)

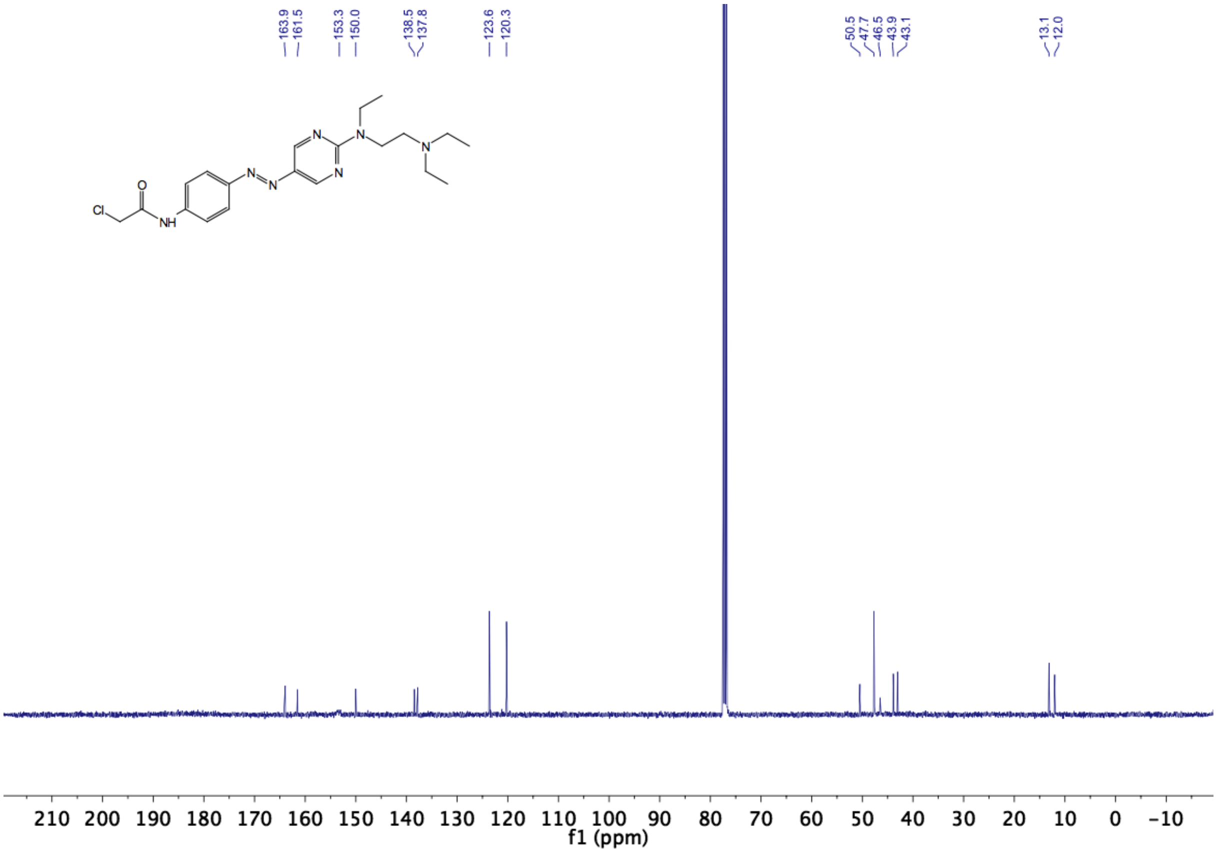
163.9
161.5
153.3
150.0
138.5
137.8
123.6
120.3
50.5
47.7
46.5
43.9
43.1
13.1
12.0
Cl
O
NH
N
N
N
N
N
N
f1 (ppm)

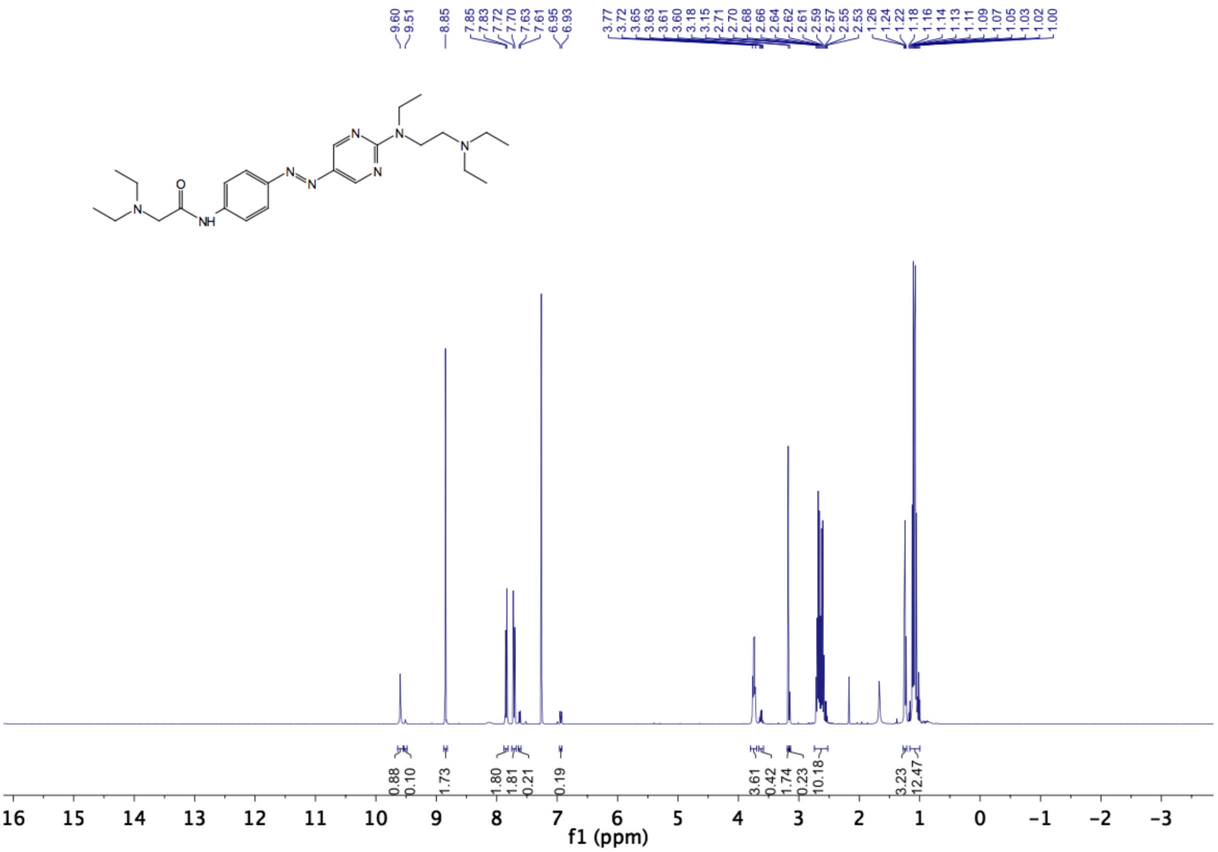
9.60
9.51
8.85
7.85
7.83
7.72
7.70
7.63
7.61
6.95
6.93
3.77
3.72
3.65
3.63
3.61
3.60
3.18
3.15
2.71
2.70
2.68
2.66
2.64
2.62
2.61
2.59
2.57
2.55
2.53
1.26
1.24
1.22
1.18
1.16
1.14
1.13
1.11
1.09
1.07
1.05
1.03
1.02
1.00
0.88
0.10
1.73
1.80
1.81
0.21
0.19
3.61
0.42
1.74
0.23
10.18
3.23
12.47
f1 (ppm)

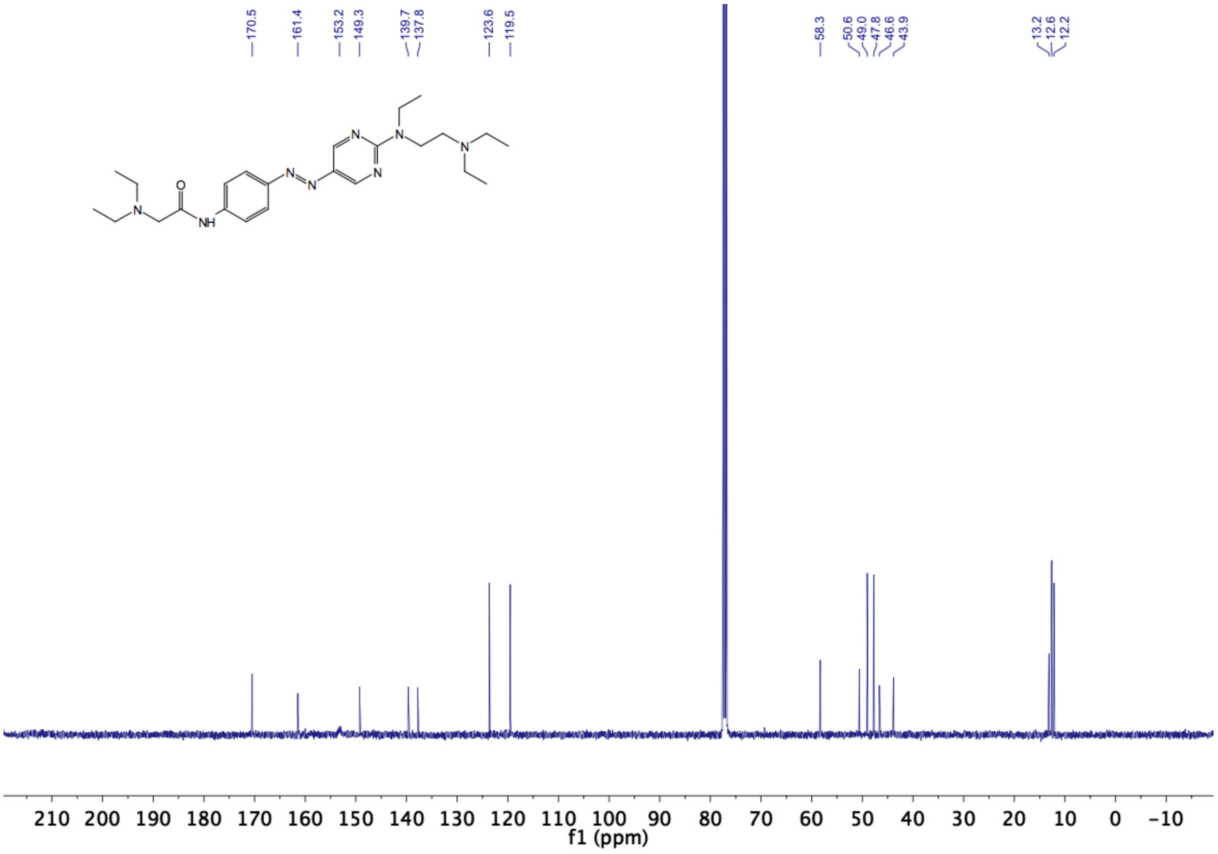
170.5
161.4
153.2
149.3
139.7
137.8
123.6
119.5
58.3
50.6
49.0
47.8
46.6
43.9
13.2
12.6
12.2
f1 (ppm)

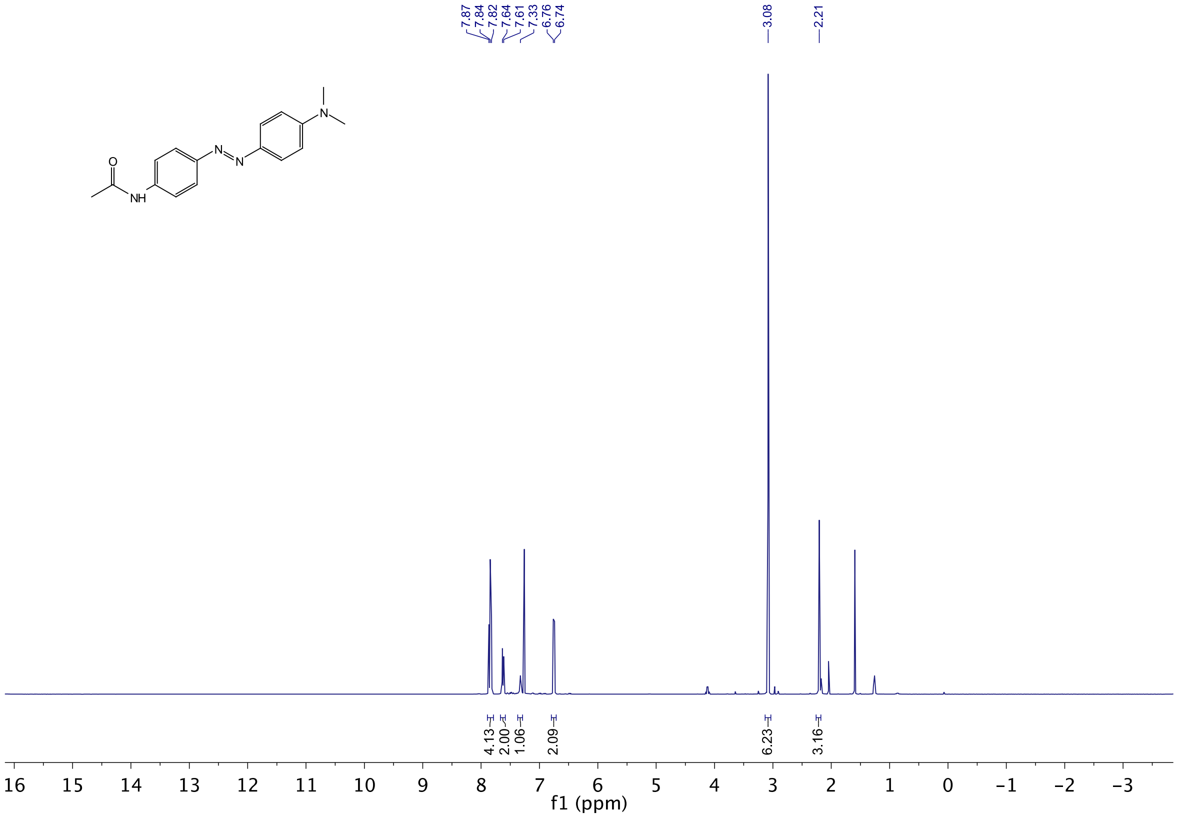
7.87
7.84
7.82
7.64
7.61
7.33
6.76
6.74
3.08
2.21
O
NH
N
N
N
4.13
2.00
1.06
2.09
6.23
3.16
16 15 14 13 12 11 10 9 8 7 6 5 4 3 2 1 0 −1 −2 −3
f1 (ppm)

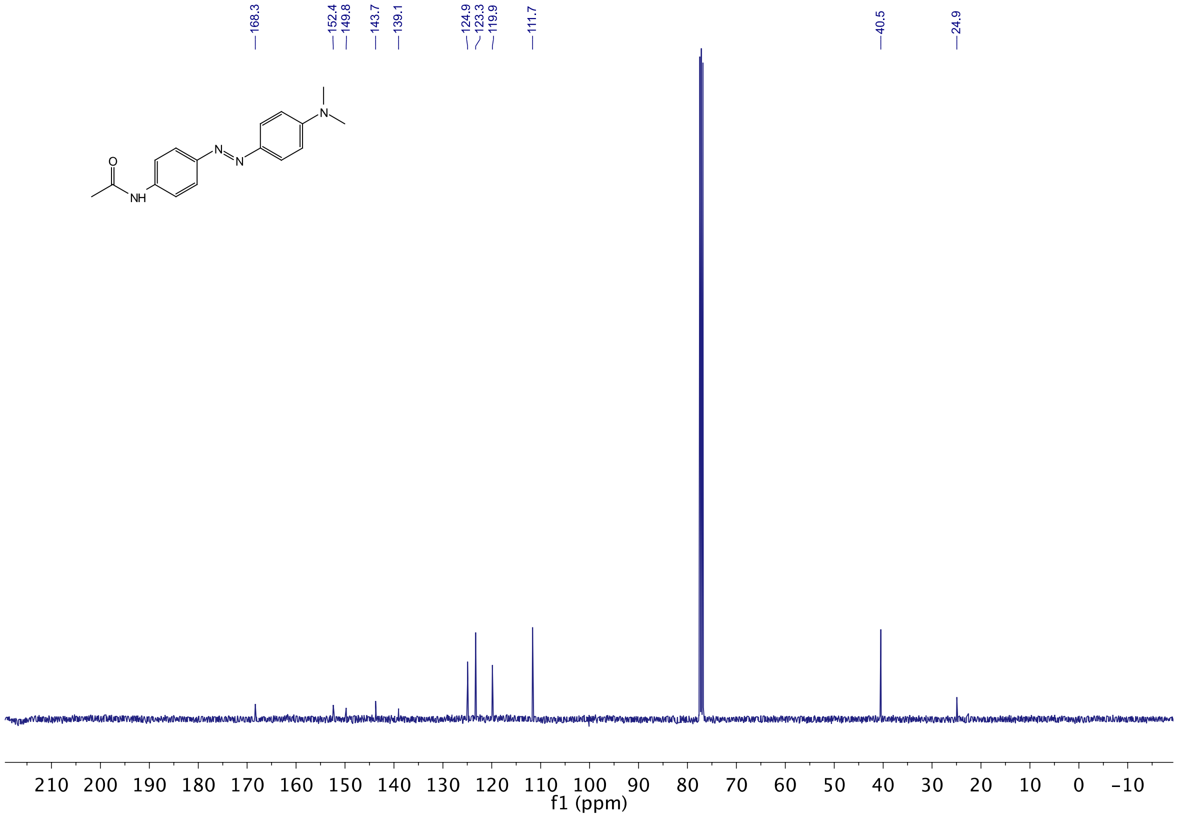
168.3
152.4
149.8
143.7
139.1
124.9
123.3
119.9
111.7
40.5
24.9
O
NH
N
N
N
210 200 190 180 170 160 150 140 130 120 110 100 90 80 70 60 50 40 30 20 10 0 −10
f1 (ppm)

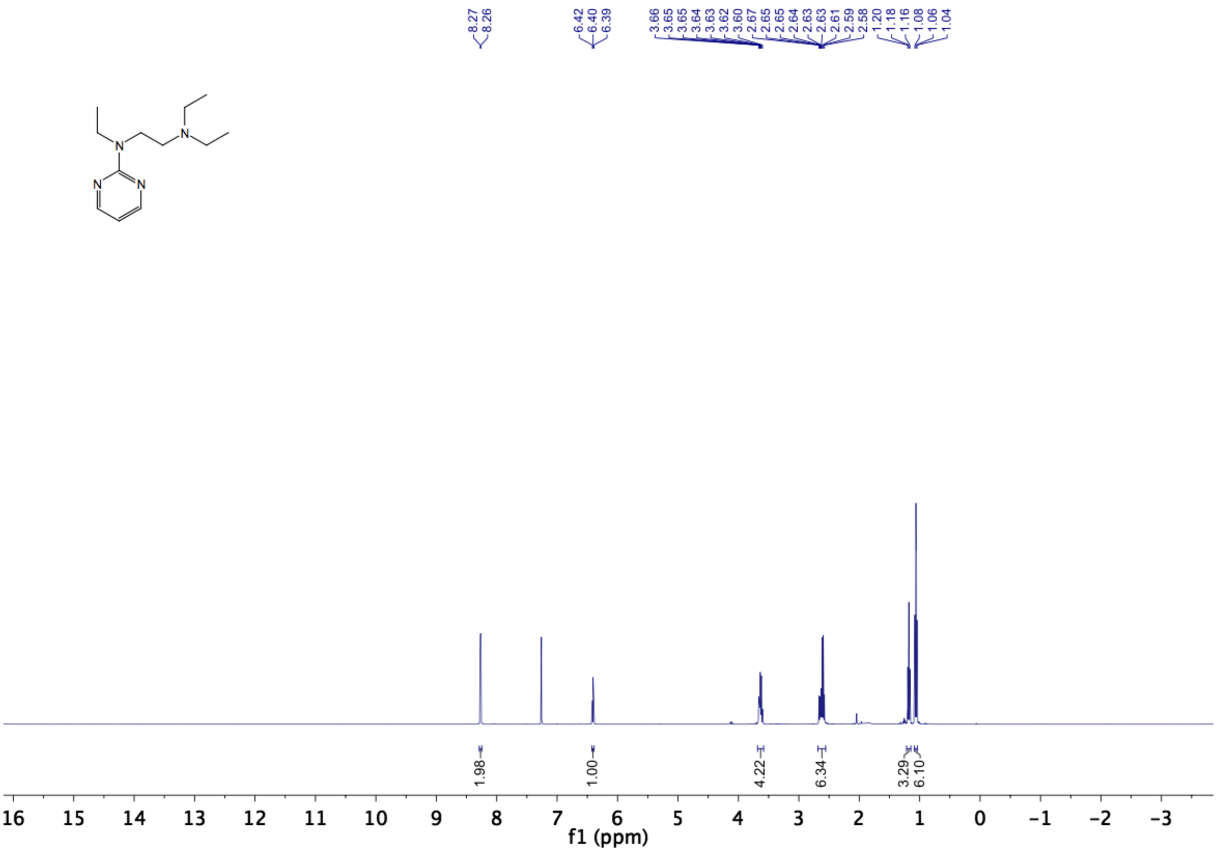
8.27
8.26
6.42
6.40
6.39
3.66
3.65
3.65
3.64
3.63
3.62
3.60
2.67
2.65
2.65
2.64
2.63
2.63
2.61
2.59
2.58
1.20
1.18
1.16
1.08
1.06
1.04
1.98
1.00
4.22
6.34
3.29
6.10
f1 (ppm)

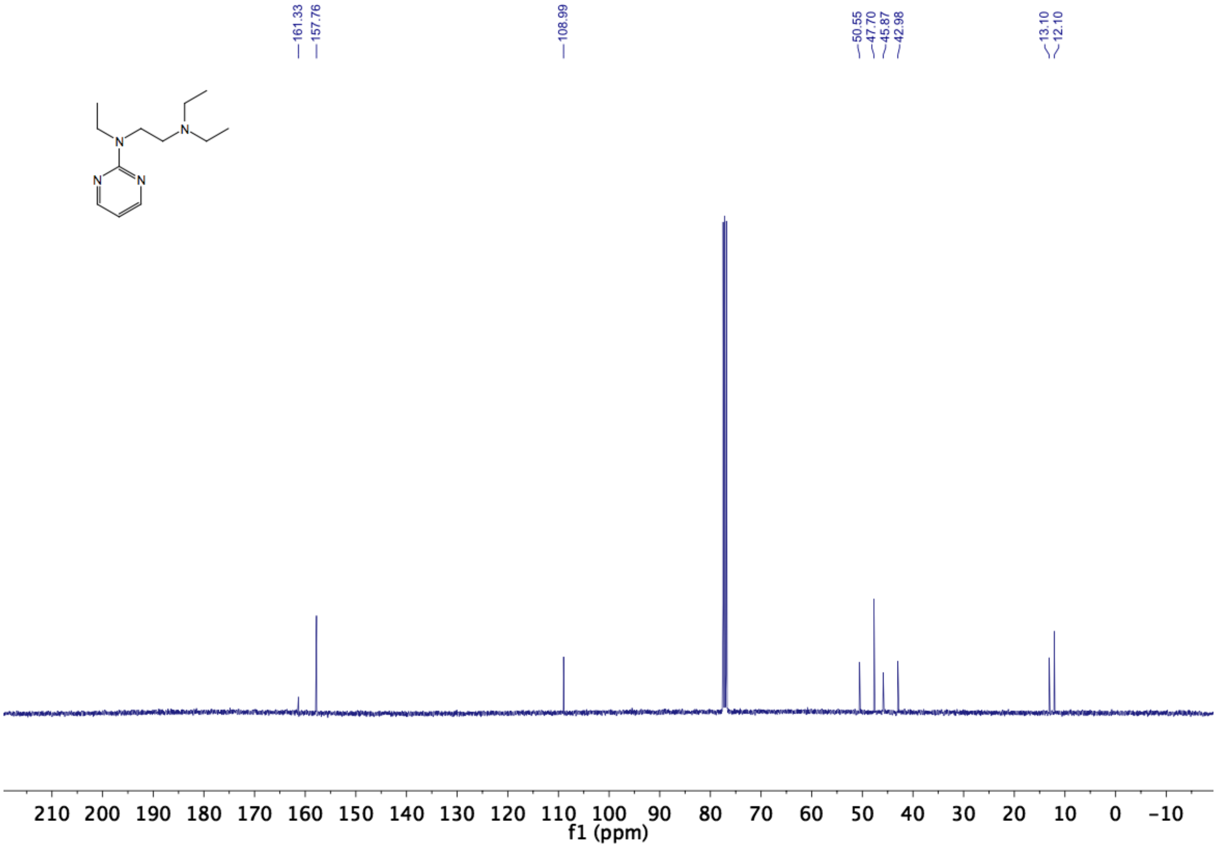
161.33
157.76
108.99
50.55
47.70
45.87
42.98
13.10
12.10
f1 (ppm)

Supplementary References

1. Martínez, C. H. R. & Dardonville, C. Rapid Determination of Ionization Constants (pKa) by UV Spectroscopy Using 96-Well Microtiter Plates. *ACS Med. Chem. Lett.* **4**, 142–145 (2013).
2. Laprell, L. *et al.* Photopharmacological control of bipolar cells restores visual function in blind mice. *J. Clin. Invest.* **127**, (2017).
